# Dynamic Metabolomic Changes in the Phenolic Compound Profile and Antioxidant Activity in Developmental Sorghum Grains

**DOI:** 10.1021/acs.jafc.4c08975

**Published:** 2024-12-30

**Authors:** Carolina Thomaz Dos Santos D’almeida, Marie-Hélène Morel, Nancy Terrier, Hamza Mameri, Mariana Simões Larraz Ferreira

**Affiliations:** 1 Laboratory of Bioactives (LABBIO), Food and Nutrition Graduate Program (PPGAN), Federal University of the State of Rio de Janeiro (UNIRIO), Rio de Janeiro 22290-240, Brazil; 2 Center of Innovation in Mass Spectrometry, Laboratory of Protein Biochemistry, UNIRIO, Rio de Janeiro 22290-240, Brazil; 3 UMR 1208 IATE, Univ Montpellier, INRAE, L’Institut-Agro Montpellier, Montpellier F-34060, France; 4 AGAP Institute, Univ. Montpellier, INRAE, CIRAD, Montpellier F-34398, France

**Keywords:** antioxidant compounds, polyphenols, *Sorghum bicolor*, UHPLC-MS^E^

## Abstract

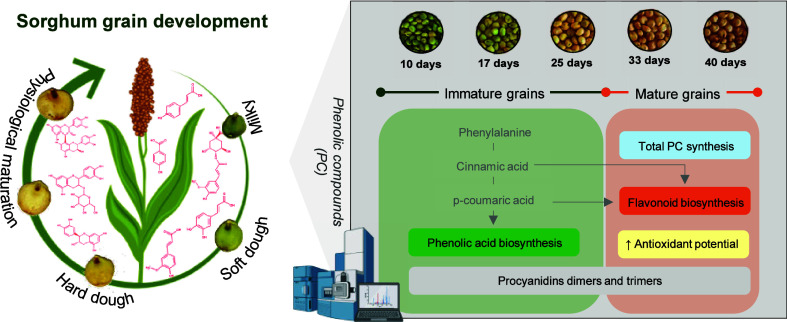

Phenolic compounds (PC) were analyzed by UHPLC-ESI-QTOF-MS^E^ in two sorghum genotypes, harvested in two growing seasons (GS) at five distinct days after flowering (DAF) to evaluate how genotype/GS influences the PC synthesis and antioxidant capacity during grain growth. Total phenolic contents were strongly correlated with antioxidant capacity (*r* > 0.9, *p* < 0.05). Globally, 97 PC were annotated, including 20 PC found irrespective of the grain developmental stage and genotype/GS. The phenolic profile clearly differs between stages: phenolic acids were the most abundant class in early stages (50%), and flavonoid accumulation becomes predominant in late ones (3/5 of total ion abundance). Dimeric and trimeric tannins were identified even in 10DAF grains. Chemometry revealed great PC variability between genotypes (27%) and important biomarkers of GS differentiation (e.g., ferulic acid). This work can input open databases of PC and paves the way to understand biosynthetic pathways of PC in sorghum and future sorghum selection

## 1 Introduction

Sorghum, botanically known as *Sorghum bicolor* L. Moench, is considered the fifth most important carbohydrate-rich crop in the world. Despite sorghum being considered a staple food for about 500 million people in 30 countries in Africa and Asia, most of the sorghum is used as animal feed in almost all western countries.^2^ Due to climate oscillations, the use of sorghum in human nutrition has been required since this cereal can support drastic agronomic and environmental circumstances. With its resilience to low rainfall, low availability of water for irrigation and salinity, this cereal offers a possible solution to food production stagnation and can ensure world food security.^3^ In addition to its agronomic advantages, sorghum grain is a rich source of nutrients,^4^ and most importantly, contains a diverse range of health-promoting bioactive phenolic compounds.^5^

Phenolic compounds (PC) are a group of specialized metabolites, naturally biosynthesized by plants to act as defense agents in response to possible stresses caused during their development. These compounds are associated with diverse human health benefits, such as reducing oxidative stress (antioxidant capacity) and the growth of various cancer cells, including colon, hepatoma, esophageal, intestinal epithelial, leukemia, breast, and stomach cancer cells.^6−9^ In sorghum, PC are concentrated in the grain outermost layers (bran), and they have a diversified profile, with the classes of flavonoids (such as flavonols, flavones, flavanones, and tannins) and phenolic acids being the most abundant.^4^

Comprehensive knowledge about these compounds is the prerequisite for its industrial applications and classification; e.g., sorghum has been traditionally classified according to its tannin contents into high- and low-tannin sorghum. Tannins are positively related to reduced postprandial blood glucose release,^1,10^ reducing the caloric value of starchy foods^11^ and high antioxidant capacity.^12^ However, high-molecular-weight condensed tannins are known to bind with proteins, severely limiting their bioacessibility and digestibility.^13^ The profile and levels of tannins and others PC depend on the genotype, pedoclimatic, and growth conditions.

The sorghum plant develops in a predictable manner characterized by three distinct growth stages: vegetative growth, panicle initiation, and grain filling. The latter stage begins with flowering and continues until dry matter accumulation (physiological maturity or when grain attains the maximum dry weight). Tannin and other PC synthesis begins at this stage (60–90 days after sorghum crop planting).^14^ Sorghum grain development progresses from milky to physiological maturity over 25 to 45 days after flowering (DAF), depending on the genotype and environmental conditions.^15^ Although the variation in macronutrient composition during this process is well established in the literature,^15^ the PC synthesis during the development process is largely unknown.

Recent advances in the metabolomics field have contributed to a better understanding of plant metabolism; metabolome analyses in crop science can provide valuable information that goes beyond biomarker identification to a tool for discovering active drivers involved in biological processes.^16^ In this study, we aimed to investigate the temporal changes in the sorghum grain phenolic profile and antioxidant capacity of different genotypes and growing seasons at five developmental stages. These results provide insights into the PC biosynthesis in sorghum during grain development.

## 2 Materials and Methods

### 2.1 Chemicals and Reagents

The following reference standards, as well as MS-grade acetonitrile and methanol, were purchased from Sigma–Aldrich (St. Louis, MO, USA): vanillic acid, *p*-coumaric acid, catechin, caffeic acid, ellagic acid, *trans*-ferulic acid, kaempferol, myricetin, pyrogallol, flavanone, quercetin, gallic acid, epicatechin, 4-hydroxybenzyl alcohol, 4-hydroxyxy benzaldehyde acid, 4-hydroxybenzoic acid, 4-hydroxybenzoic acid, 4-phenylacetic acid, synapinic acid, benzoic acid, quercetin 3 glycoside, 3,4-diOH phenylacetic acid, epigallocatechin, epigatechin gallate, chlorogenic acid, 2,5-dihydroxy benzoic acid, 4-methoxycinnamic acid, 2-hydroxycinnamic acid, 3-hydroxy-4-methoxycinnamic acid, *trans-*cinnamic acid, 3-methoxycinnamic acid, and l-(−)-3 phenylacetic acid. Formic acid was purchased from Fluka (Switzerland). Milli-Q water was obtained through a Barnstead Smart2Pure (Thermo Fisher Scientific, USA) purification system. Other unmarked reagents were of analytical grade.

### 2.2 Samples

Immature sorghum (*S. bicolor* L., caudatum race) grains from two different genotypes: (1) red pericarp (IS15752, pigmented testa and presence of condensed tannins) and (2) white pericarp (Macia, without pigmented testa and tannin-free), were cultivated and collected by the unit “Genetic Improvement and Adaptation of Tropical and Mediterranean Plants” (UMR-AGAP, CIRAD, INRAE, Montpellier, France). Two experiments were conducted: in summer 2017 at Mauguio (GS1 for Macia and IS15752, southern France: 43°36′43″N, 3°58′2′′ E) and in summer 2018 at Lavalette (GS2 for Macia, southern France: 43°38′45.366′′ N, 3°52′10.218′′ E), in the field and under rainfed conditions with supplementary irrigation (Supplementary Figure 1A). Each plot consisted of three raws. Raws were spaced 0.8 m, were 5 m long, and contained 64 plants.

The grains were collected at five stages, namely, 10DAF, 17DAF, 25DAF (grain filling stage), 33DAF (dough stage), and 40DAF (harvest maturity stage) (Supplementary Figure 1A); for each stage, three replicates were collected, and each replicate consisted of three panicles of independent randomly selected plants. Panicles were stored at −60 °C after harvest. Whole grains were freeze-dried at 12% moisture content and cryogenically ground for 2 min using a ball mill, and the resulting powder was maintained at −80 °C until analysis.

### 2.3 Free and Bound Compound Extraction

To obtain free and conjugated PC from sorghum flour, the extraction technique according to Santos et al.^17^ was performed in triplicate with some modifications. Free phenolic compounds (FPC, soluble) were extracted in 80% ethanol at a ratio of 1:20 (w/v) and stirred at room temperature (25 °C, 200 rpm) for 10 min. After 10 min of centrifugation at 5000*g* at 25 °C, the supernatant was removed and stored in Eppendorf tubes (−80 °C). Extraction was performed twice, and the extracts obtained were pooled. The pellets resulting from FPC extraction were submitted to alkaline hydrolysis with 1:70 (w/v) of 4 M NaOH (submerged in an ultrasonic bath, 42 kHz, 90 min, 40 °C). After, acid hydrolysis was performed with concentrated HCl (∼pH 2), and the samples were centrifuged (2000*g,* 5 min). The supernatant was washed three times with ethyl acetate (7 mL) and centrifuged between each wash step (10,000*g*, 5 min, 10 °C) to obtain the bound phenolic compounds (BPC, insoluble). Both extracts (FPC and BPC) were evaporated (SpeedVac Savant, ThermoFisher Scientific, USA) and reconstituted in 500 μL of methanol, acetonitrile, and Milli-Q water (2:5:93, v/v/v). The reconstituted extracts were filtered (0.22 μm, hydrophilic PTFE, Analytical) and stored in vials at −80 °C.

### 2.4 Total Reducing Capacity

The total reducing capacity (TRC) was determined by a Folin–Ciocalteu method, in triplicate, according to Singleton et al.,^18^ adapted for microplates. Extracts (100 μL) were added to 700 μL of Milli-Q water in test tubes. After homogenization, 50 μL of Folin–Ciocalteu reagent and 150 μL of 20% sodium carbonate were added. The mixture was incubated (30 min, 40 °C), and 300 μL of the final solution was transferred to a microplate. The absorbance reading at 750 nm was performed in a FlexStation III microplate reader (Molecular Devices). Solvent blank and standard curve analyses were performed with gallic acid (5 to 130 μg/mL). Results were expressed in milligrams of gallic acid equivalents (GAE) per 100 g of sample, in dry basis.

### 2.5 Determination of Antioxidant Capacity

The antioxidant capacity of samples was determined, in triplicates, by the DPPH (2,2-diphenyl-1-picrylhydrazyl) radical scavenging method and the ferric-reducing antioxidant power method (FRAP), adapted to microplates.^19^ For the DPPH method, a 20 μL aliquot of each extract was combined with 280 μL of the DPPH solution (32 μg/mL) and the mixture was incubated (30 min, in the dark, 25 °C). For FRAP assays, the reagent was prepared in acetate buffer (0.3 M, pH 3.6), FeCl_3_·6H_2_O (20 mM), and TPTZ solution (10 mM) in a 10:1:1 ratio. A 20 μL aliquot of each extract was combined with 15 μL of Milli-Q water and 265 μL of FRAP reagent, gently vortexed, and incubated (30 min, 37 °C). Absorbance was measured using a microplate reader (FlexStation III, Molecular Devices, USA) at 715 and 595 nm, respectively, and results were expressed as μmol of trolox (6-hydroxy-2,5,7,8-tetramethylchroman-2-carboxylic acid) equivalents (TE) per 100 g of sample, in dry basis.

### 2.6 Metabolomic Analysis of Sorghum Grains by UHPLC-MS^E^

The phenolic profiling was performed by injecting 5 μL of each sample into an Ultrahigh-Performance Liquid Chromatography (UHPLC) Acquity system (Waters, USA) coupled with a XEVO G2S Q-Tof (Waters, England) equipped with ionization source electrospray. An UHPLC HSS T3 C18 column (100 × 2.1 mm, 1.8 μm particle diameter; Waters) at 30 °C and flow rate of 0.5 mL/min of ultrapure water containing 0.3% formic acid and 5 mM ammonium formate (mobile phase A) and acetonitrile containing 0.3% formic acid (mobile phase B) was used according to the following gradient method: 0 min -97% A; 11.80 min -50% A; 12.38 min -15% A; 14.11–97% A. Data were acquired in triplicate in MS^E^ negative and centroid mode between *m*/*z* 50 and 1200, collision energy ramp from 30 to 55 V, cone voltage 30 V, capillary voltage 3.0 kV, desolvation gas (N_2_) 1200 L/h at 600 °C, cone gas 50 L/h, source at 150 °C, and using leucine enkephalin (Leu-Enk, *m*/*z* 554.2615, [M-H]-) for calibration. A mix containing 33 analytical standards of phenolic compounds (10 ppm) was prepared and injected in triplicate, prior to the injection of the samples, to ensure the reproducibility of the instrument and to confirm phenolic compound identification. Besides the injection of the chemical standards, a set of quality control (QC) samples was also prepared by pooling equal volumes of each sorghum extract and were injected after each batch of six runs of sorghum samples to monitor the instrument’s stability.

MassLynx v 4.1 software (Waters, USA) was used to acquire MS data, and Progenesis QI (Waters, USA) software was applied to data processing. Nontargeted identification was performed according to Metabolomics Initiative Standard as described by Sumner et al.^20^ considering a customized database built from PubChem and online database Phenol-Explorer. The metabolite identification (level 1) was based on standard run parameters, such as isotope distribution of neutral mass, exact mass, retention time, and MS/MS fragments spectra. The following parameters were applied to annotated metabolites (levels 2 and 3): exact mass error (<10 ppm), isotopic similarity (>80%), score (>30), and the highest score of fragmentation, generated by the software. Data from the literature and chemical characteristics of the molecules were also used to help the tentative annotation of unknown compounds. In addition, only compounds present in the three technical replicates and showing CV < 30% were considered. The resulting compounds had their normalized relative abundance divided by one hundred and multiplied by the average grain dry weight (mg DM·grain^–1^) to calculate the relative phenolic abundance per grain. Metabolic pathways were proposed based on the observed phenolic changes in this study and their comparison with the KEGG phenylpropanoid biosynthesis pathway (map00940).

### 2.7 Statistical Analysis

Statistical analysis was performed with a Tukey’s test (*p* < 0.05) and one-way ANOVA, using XLSTAT software (Addinsoft, France). The raw data obtained by UHPLC-MS^E^ was normalized by total ion count (TIC) using Progenesis QI software, where each metabolite’s intensity obtained from the ion sample mass spectra is divided by the total intensity (sum of all ion intensities) observed in the sample. Data generated were exported to perform multivariate analysis such as hierarchical cluster analysis by XLSTAT and orthogonal partial least-squares discriminate analysis (OPLS-DA) by EZinfo 3.0. The efficiency and reliability of the OPLS-DA models were verified by percent variation of the *y* variables explained by the model (R2Y) and the predictive performance of the model (Q2) using the Metaboanalyst 5.0 web server (https://www.metaboanalyst.ca/). In addition, permutation tests were carried out with 100 random permutations to validate the OPLS-DA models.

## 3 Results and Discussion

### 3.1 Evaluation of Sorghum Grain Development

Supplementary Figure 1 presents the images of the sorghum grains and their respective growth curves. Visible morphological changes (Supplementary Figure 1A) and the sigmoidal growth curve of the average grain weight analyzed across contrasting genotypes and growing seasons (GS) (Supplementary Figure 1A) clearly delineated the filling stages of the sorghum grain. Both genotypes and GS demonstrated rapid growth during the initial grain development stages (10–25DAF), indicative of rapid grain filling postflowering. By 33DAF, the grains reach their maximum weight, followed by a stabilization phase at 40DAF.

The sorghum grain formation involves four stages: (1) milky stage, initial stage of grain development that occurs around 10DAF; (2) soft and (3) hard dough stages, stages where the grain reaches about 50 and 75% of its total dry weight, respectively; and (4) physiological maturity, indicating that maturation is complete after full grain filling (100% of its total dry weight).^15^ In the present study, all these stages of grain development were covered and would be effective in understanding PC synthesis during sorghum grain maturation: 10DAF = milky stage; 17DAF = soft dough stage; 25DAF = hard dough stage; and 33 and 40DAF = physiological maturity (Supplementary Figure 1B).

Similarities and differences between genotypes and GS (two crop years) during grain formation were observed. While for the Macia genotype, there was no variation: Macia GS1 and Macia GS2 are statistically equal in all stages, showing similar profiles of grain dry mass accumulation; the IS15752 genotype showed a peculiar behavior in some stages (*p* < 0.05) (Supplementary Figure 1B): at 17DAF it showed lower grain dry mass values than Macia GS1 and GS2 (−36 and −44%, respectively), while at 33DAF, these values were higher (+21 and +14%, respectively), suggesting that the conversion of sugars and amino acids into starch and protein, respectively, may occur later in IS15752, but its effective filling in later stages forms heavier grains.

### 3.2 Phenolic Compounds and Antioxidant Capacity

**Figure 1:**
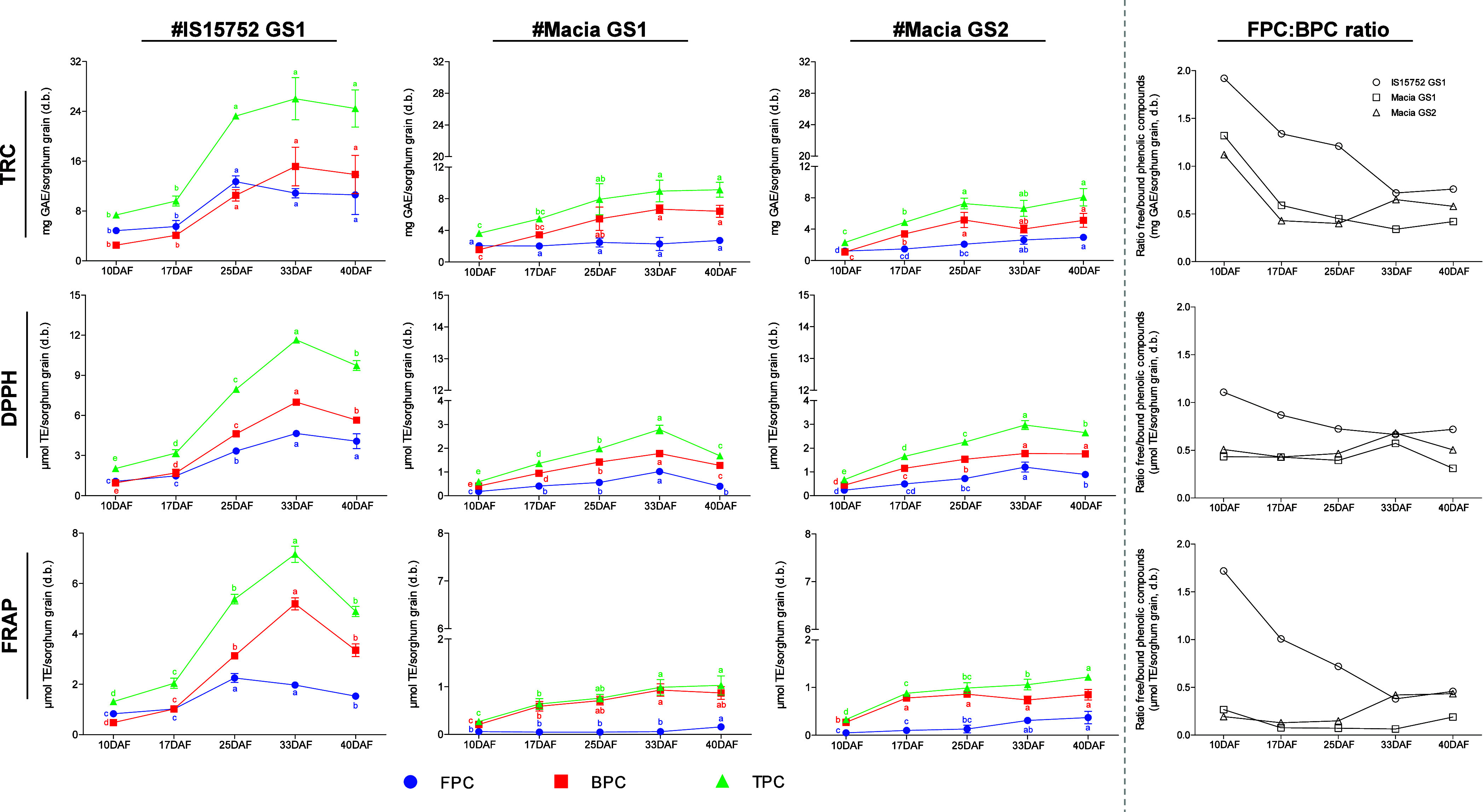
Evaluation of days after flowering (DAF) in total reducing capacity (TRC) and antioxidant capacity (DPPH and FRAP methods) in free (FPC), bound (BPC), and total (TPC) phenolic compound extracts in different genotypes and growing seasons (GS) of sorghum grains. The ratio between the FPC and BPC values in each analysis is shown in the last column. Results are expressed as mean ± standard deviation (*n* = 3). Different letters indicate a significant difference between DAF (Tukey, *p* < 0.05).

The TRC was determined in both free (FPC) and bound (BPC) extracts throughout grain development in the two genotypes and two GS (Figure 1). IS15752 GS1 ranged from 7.38 ± 0.29 to 26.03 ± 3.39 for the total extract (TPC corresponds to the sum of FPC and BPC), and, in general, the values at each stage were 3-fold greater than that found in Macia GS1 (3.62 ± 0.29 to 9.13 ± 0.94) and GS2 (2.30 ± 0.19 to 8.07 ± 1.11). The higher values observed for IS15752 in all antioxidant analysis (*p* < 0.05) can be explained by the presence of tannins in this genotype.^21^ When comparing the different GS (for the Macia genotype), the most immature stage (10DAF) showed TRC values 57% higher in GS1 when compared to GS2 (*p* < 0.01), while in the other stages, the values were similar. The hypothesis is that the Macia GS1 genotype underwent some abiotic particular conditions in the initial stage of grain growth, which favored the synthesis of PC to protect plants from oxidative stress.^22^

The changes of TRC throughout grain development behaved in a similar way in all samples, showing a progressive increase toward maturation. The initial stages (10DAF and 17DAF) have lower TRC values, followed by a significant increase in 25DAF (142, 46, and 49% in IS15752 GS1, Macia GS1, and Macia GS2, respectively, compared to 10DAF), and constant values in mature stages (33 and 40DAF) (Figure 1). In Macia genotypes, this increase was essentially due to the BPC, mainly in GS1, while for the IS15752 genotype, the significant increase occurred until 25DAF for both FBC and BPC and then stabilized. In contrast to what has been reported in wheat grain,^17^ this result indicates an insolubilization and complexation of phenolic compounds during sorghum grain development. Indeed, the FPC:BPC ratio progressively reduced during grain development for IS15752 GS1 (from 1.92 to 0.76), Macia GS1 (from 1.32 to 0.42), and Macia GS2 (from 1.12 to 0.58), suggesting that BPC are the main responsible for the TPC increase. These results corroborate previously published data with maize kernels;^23^ however, in this case, FPC is the main responsible for the increase, highlighting a dissimilarity between sorghum and maize.

Looking at each extract (free and bound) separately, the FPC extract was predominant in the Macia genotype at 10DAF (57% of TPC), but the significant synthesis of BPC in the soft dough stage makes it the majority (BPC averaged 54% of TPC from 25DAF). During *in vivo* digestion, BPC reach the colon and are processed/transformed by microbial activity, presenting potential beneficial effects on human health.^24^ In IS15752 GS1, the BPC start to be produced (synthesized and linked to other components) early in the grain development and show the maximum by 25–33DAF. This result is expected for this genotype since it is classified as high condensed tannins (procyanidins), and this phenolic class is usually bound to components of the plant matrix.^4^ Despite this, it is important to consider that although the interactions between condensed tannins and other matrix components can be broken by the action of acid hydrolysis, the method applied in the present study was not efficient to depolymerize and consequently to extract and to quantify these compounds; also, the Folin–Ciocalteu method present interferences with other reducing power substances such as ascorbic acid, aromatic amines, and sugars.

As expected, the antioxidant capacity measured by DPPH and FRAP methods showed a strong correlation with TRC (0.9846 and 0.9737, *p* < 0.05, respectively). Through these different methods, it is possible to observe that, although the general behavior during sorghum grain development is similar between genotypes and GS, there are variations in their proportions in the extracts (free and bound). In Macia genotypes, irrespective to GS, BPC extract was almost superposed to TPC in FRAP and DPPH results. Since IS15752 GS1 had the same growing conditions as Macia GS1, it is believed that these variations can be associated with the proanthocyanidin-rich composition of the first genotype.

### 3.3 Identification of Phenolic Compounds by UHPLC-MS^E^

The phenolic profiles of the different sorghum genotypes and GS were followed during different grain development stages by the UHPLC-MS^E^ method, providing the most comprehensive screening in sorghum grains to date. Globally, a total of 97 PC were tentatively identified; among them, 11 compounds were fully confirmed by reference standards (Supplementary Table 1, compounds in bold): 7 were present in both extracts (free and bound); 2 were identified only in free extract; and 2 were identified only in bound extract. Contrary to findings in the literature, which indicated that the number of compounds identified in immature cereal grain samples and mature whole sorghum samples was greater in bound extracts compared to free extracts,^17,21,25^ our study found that the majority of PC were present in free extracts (exclusive 47 PC) rather than in bound extracts (exclusive 32 PC). A total of 18 PCs were commonly identified in both extracts. The predominance of FPC is also observed in the total relative ion abundance, where the abundance of FPC was 112% higher than BPC. The annotated phenolic compounds belonged mainly to the flavonoid class (54%), followed by phenolic acids (32%), other polyphenols (12%), and lignans (2%). One compound ([M–H]^−^ 10.16 *m*/*z* 191.0343) could not be assigned to a class and was classified as an unknown compound.

**Figure 2:**
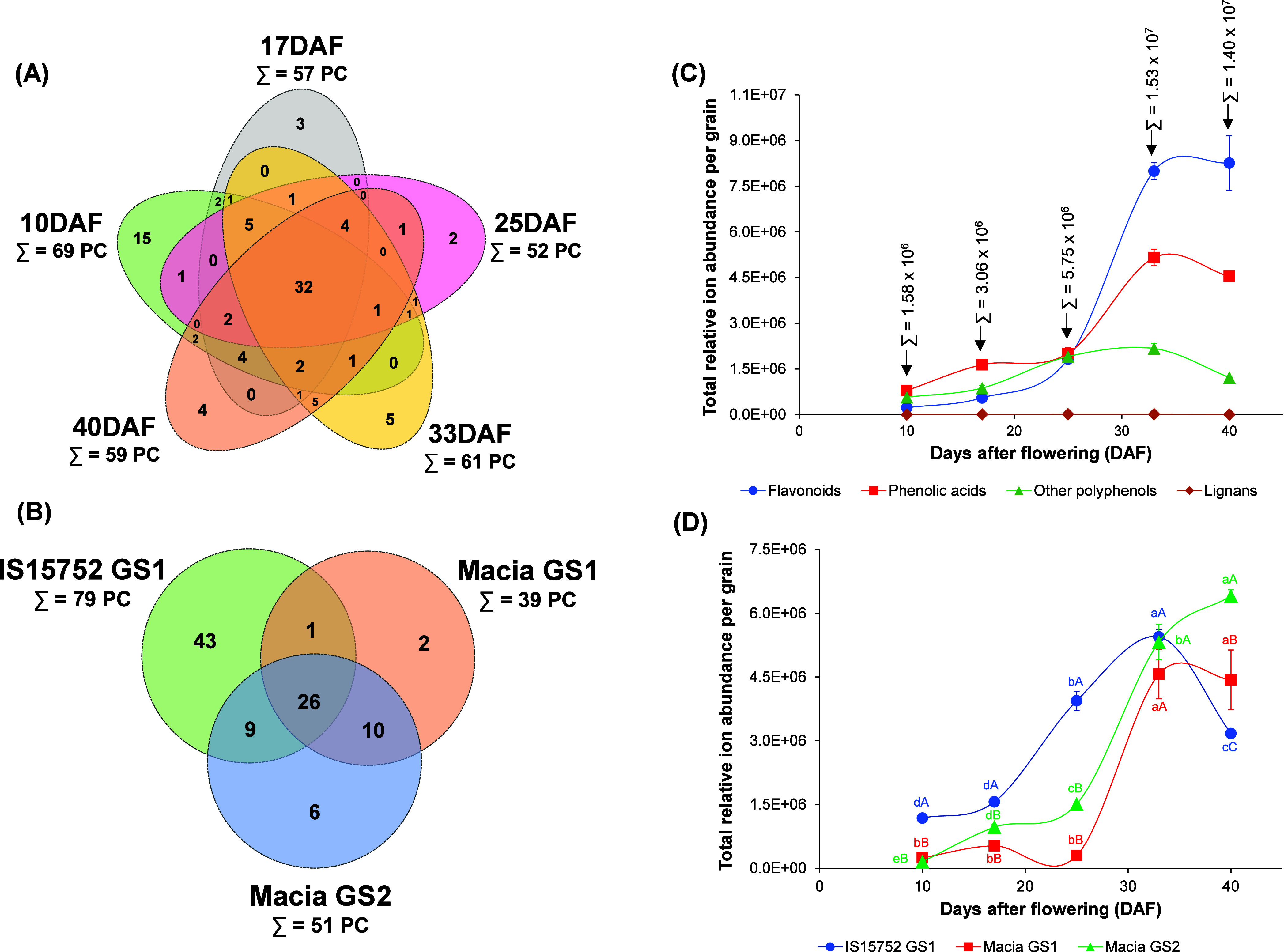
Metabolomic analysis. (A) Venn diagram with the number of identification distribution in grains from different development stages. (B) Total relative ion abundance of phenolic compounds by class during grain development. (C) Total relative ion abundance of phenolic compounds in each sample during DAF. (D) Venn diagram with the number of identification distribution in each genotype/growing season (GS). Σ = sum of the total group value. Different lowercase and uppercase letters mean a significant difference (*p* < 0.05 by one-way ANOVA and Tukey post-test) between DAF and samples/genotypes, respectively. Bars represent standard deviation (*n* = 3).

Some compounds were systematically present independent of the development stage (32 PC) or the genotype and GS (26 PC) (Figure 2A,B, intersections of the Venn diagram). These compounds and their relative abundance by total ion counting are described in Table 1, where the confirmed identifications with phenolic patterns are in bold. Additionally, among these compounds, 20 PC were common across all samples regardless of the developmental stage or genotype/GS (Table 1, compounds marked with an asterisk).

**Table 1:** Phenolic Compounds (PC) Found in Common between the Different Developmental Stages Indicated by Days after Flowering (DAF, *n* = 32) or between Genotypes/Growing Season (GS, *n* = 26) of Sorghum Grains (per Seed)^a^

					IS15752 GS1					**Macia GS1**					**Macia GS2**				
**name of compound**	**molecular formula**	***m/z***	**RT (min)**	**class**	**10DAF**	**17DAF**	**25DAF**	**33DAF**	**40DAF**	**10DAF**	**17DAF**	**25DAF**	**33DAF**	**40DAF**	**10DAF**	**17DAF**	**25DAF**	**33DAF**	**40DAF**
PC in common among all days after flowering (*n* = 32)																			
4-hydroxybenzaldehyde*	**C**_**7**_**H**_**6**_**O**_**2**_	**121.0289**	**6.14**	**OP**	4.52 × 10^5^	6.97 × 10^5^	1.67 × 10^6^	1.58 × 10^6^	0	4.46 × 10^4^	8.09 × 10^4^	8.80 × 10^4^	7.94 × 10^4^	6.10 × 10^4^	5.35 × 10^4^	4.19 × 10^4^	8.18 × 10^4^	0	2.22 × 10^5^
caffeic acid*	**C**_**9**_**H**_**8**_**O**_**4**_	**179.0343**	**6.24**	**PA**	9.42 × 10^4^	8.05 × 10^4^	1.81 × 10^5^	4.14 × 10^5^	5.29 × 10^5^	1.17 × 10^4^	3.26 × 10^4^	0	4.46 × 10^5^	6.08 × 10^5^	2.02 × 10^4^	4.08 × 10^4^	0	5.10 × 10^5^	3.63 × 10^5^
*trans*-ferulic acid*	**C**_**10**_**H**_**10**_**O**_**4**_	**193.0499**	**8.00**	**PA**	6.70 × 10^4^	1.15 × 10^5^	1.49 × 10^5^	2.28 × 10^5^	1.99 × 10^5^	9.20 × 10^4^	1.92 × 10^5^	2.87 × 10^4^	4.21 × 10^5^	3.43 × 10^5^	4.54 × 10^3^	3.30 × 10^5^	4.53 × 10^5^	3.53 × 10^5^	3.47 × 10^5^
*p*-coumaric acid*	**C**_**9**_**H**_**8**_**O**_**3**_	**163.0393**	**7.48**	**PA**	2.51 × 10^4^	5.07 × 10^4^	1.28 × 10^5^	6.56 × 10^4^	2.38 × 10^5^	6.97 × 10^3^	1.01 × 10^5^	5.60 × 10^4^	2.88 × 10^5^	4.68 × 10^4^	1.03 × 10^4^	1.31 × 10^5^	4.48 × 10^4^	3.20 × 10^5^	4.95 × 10^5^
scutellarein*	C_15_H_10_O_6_	285.0392	10.16	F	8.49 × 10^2^	1.21 × 10^4^	1.66 × 10^4^	0	1.76 × 10^3^	0	2.54 × 10^4^	0	0	2.96 × 10^5^	0	4.65 × 10^4^	1.71 × 10^5^	4.09 × 10^5^	5.54 × 10^5^
3′-hydroxymelanettin*	C_16_H_12_O_6_	299.0551	11.03	F	2.98 × 10^2^	0	4.96 × 10^3^	0	0	5.83 × 10^3^	1.03 × 10^3^	2.45 × 10^3^	0	0	7.92 × 10^3^	3.10 × 10^4^	2.19 × 10^5^	3.35 × 10^5^	6.93 × 10^5^
esculetin*	C_9_H_6_O_4_	177.0185	6.10	OP	2.20 × 10^3^	2.00 × 10^3^	3.73 × 10^3^	1.54 × 10^4^	3.60 × 10^4^	4.56 × 10^3^	1.09 × 10^4^	0	2.50 × 10^5^	3.58 × 10^5^	4.22 × 10^3^	1.09 × 10^4^	4.64 × 10^4^	1.84 × 10^5^	2.72 × 10^5^
dihydrocaffeic acid	C_9_H_10_O_4_	181.0500	4.46	PA	6.56 × 10^4^	1.23 × 10^5^	2.74 × 10^5^	3.84 × 10^5^	3.47 × 10^5^	0	0	0	0	0	0	0	0	0	0
ferulic acid*	C_10_H_10_O_4_	193.0498	8.25	PA	3.19 × 10^4^	2.05 × 10^3^	7.31 × 10^3^	9.90 × 10^4^	9.43 × 10^4^	4.36 × 10^4^	3.70 × 10^3^	9.57 × 10^3^	0	1.41 × 10^5^	0	1.65 × 10^5^	2.54 × 10^5^	1.55 × 10^5^	1.68 × 10^5^
naringenin 7-*O*-glucoside*	C_21_H_22_O_10_	433.1128	7.84	F	1.33 × 10^4^	2.52 × 10^4^	5.29 × 10^4^	8.95 × 10^4^	7.92 × 10^4^	0	0	4.75 × 10^4^	0	4.83 × 10^4^	0	3.80 × 10^4^	6.59 × 10^4^	5.78 × 10^4^	6.90 × 10^4^
4-hydroxybenzoic acid*	**C**_**7**_**H**_**6**_**O**_**3**_	**137.0237**	**5.03**	**PA**	1.37 × 10^4^	3.06 × 10^4^	7.30 × 10^4^	1.83 × 10^5^	1.44 × 10^5^	8.51 × 10^3^	4.56 × 10^3^	2.79 × 10^3^	1.12 × 10^4^	1.35 × 10^4^	0	7.17 × 10^3^	4.79 × 10^3^	0	3.59 × 10^4^
dihydroxybenzoic acid isomer I*	C_7_H_6_O_4_	153.0186	3.46	PA	1.70 × 10^4^	3.39 × 10^4^	6.34 × 10^4^	1.78 × 10^5^	2.29 × 10^5^	0	0	0	2.03 × 10^3^	3.72 × 10^3^	5.68 × 10^2^	1.08 × 10^3^	0	2.76 × 10^3^	0
procyanidin dimer I	C_30_H_26_O_12_	577.1338	5.42	F	2.12 × 10^4^	3.50 × 10^4^	1.63 × 10^5^	1.86 × 10^5^	1.25 × 10^5^	0	0	0	0	0	0	0	0	0	0
procyanidin trimer I	C_45_H_38_O_18_	865.1963	5.76	F	1.23 × 10^4^	2.04 × 10^4^	9.39 × 10^4^	1.11 × 10^5^	9.14 × 10^4^	0	0	0	0	0	0	0	0	0	0
hesperidin*	C_28_H_34_O_15_	609.1881	0.57	F	4.00 × 10^3^	6.16 × 10^3^	1.17 × 10^4^	1.98 × 10^4^	2.36 × 10^4^	4.36 × 10^3^	1.53 × 10^4^	2.16 × 10^4^	2.25 × 10^4^	2.29 × 10^4^	4.74 × 10^3^	1.50 × 10^4^	1.79 × 10^4^	2.11 × 10^4^	2.21 × 10^4^
5-caffeoylquinic acid*	C_16_H_18_O_9_	353.0865	5.87	PA	7.03 × 10^4^	0	2.34 × 10^4^	1.76 × 10^4^	1.44 × 10^4^	0	3.17 × 10^3^	0	8.18 × 10^3^	0	9.82 × 10^3^	1.22 × 10^4^	9.24 × 10^3^	0	2.83 × 10^4^
3-feruloylquinic acid*	C_17_H_20_O_9_	367.1023	6.07	PA	1.17 × 104	0	1.10 × 10^4^	0	1.26 × 10^4^	1.05 × 10^4^	2.58 × 10^4^	0	1.82 × 10^4^	0	2.72 × 10^4^	2.15 × 10^4^	0	2.64 × 10^4^	1.76 × 10^4^
tetramethoxyflavone isomer III*	C_19_H_18_O_6_	341.1019	10.12	F	2.31 × 10^3^	0	0	0	7.48 × 10^3^	5.03 × 10^3^	1.16 × 10^4^	1.59 × 10^4^	2.00 × 10^4^	1.98 × 10^4^	3.09 × 10^3^	1.14 × 10^4^	1.69 × 10^4^	1.88 × 10^4^	2.86 × 10^4^
naringin 4′-*O*-glucoside*	C_21_H_22_O_10_	433.1128	8.35	F	2.82 × 10^3^	5.73 × 10^3^	1.18 × 10^4^	1.99 × 10^4^	1.58 × 10^4^	0	0	1.13 × 10^4^	1.28 × 10^4^	8.59 × 10^3^	0	8.83 × 10^3^	1.42 × 10^4^	1.20 × 10^4^	1.35 × 10^4^
vanillin*	**C**_**8**_**H**_**8**_**O**_**3**_	**151.0393**	**5.80**	**OP**	2.43 × 10^3^	4.07 × 10^3^	8.14 × 10^3^	1.44 × 10^4^	1.72 × 10^4^	3.05 × 10^3^	0	0	0	7.22 × 10^3^	0	0	0	3.38 × 10^4^	3.86 × 10^4^
dihydroxy-trimethoxyflavone isomer II	C_18_H_16_O_7_	343.0811	9.93	F	0	0	0	0	0	2.11 × 10^3^	3.70 × 10^3^	8.89 × 10^3^	2.08 × 10^4^	2.61 × 10^4^	1.61 × 10^3^	6.37 × 10^3^	1.60 × 10^4^	2.19 × 10^4^	0
tetramethoxyflavone isomer I*	C_19_H_18_O_6_	341.1019	8.30	F	2.08 × 10^3^	3.92 × 10^3^	0	7.93 × 10^3^	7.42 × 10^3^	2.40 × 10^3^	4.57 × 10^3^	0	1.45 × 10^4^	1.31 × 10^4^	0	6.56 × 10^3^	1.12 × 10^4^	1.44 × 10^4^	1.48 × 10^4^
eriodictyol 7-*O*-glucoside	C_21_H_22_O_11_	449.1073	6.95	F	2.65 × 10^3^	5.41 × 10^3^	2.07 × 10^4^	3.57 × 10^4^	2.96 × 10^4^	0	0	0	0	0	0	0	0	0	0
procyanidin dimer B-type III	C_30_H_26_O_12_	577.1335	7.48	F	4.13 × 10^3^	6.10 × 10^3^	2.15 × 10^4^	2.02 × 10^4^	1.55 × 10^4^	0	0	0	0	0	0	0	0	0	0
feruloyl glucose*	C_16_H_20_O_9_	355.1023	6.46	PA	9.20 × 10^2^	0	0	0	8.41 × 10^3^	0	6.28 × 10^3^	0	4.16 × 10^3^	0	1.59 × 10^3^	1.26 × 10^4^	2.44 × 10^4^	0	4.42 × 10^3^
quercetin 3-*O*-rutinoside	C_27_H_30_O_16_	609.1447	8.07	F	5.30 × 10^3^	7.21 × 10^3^	1.50 × 10^4^	1.48 × 10^4^	1.45 × 10^4^	0	0	0	0	0	0	0	0	0	0
dihydroxy-trimethoxyflavone isomer III	C_18_H_16_O_7_	343.0812	10.59	F	0	0	0	0	0	0	0	0	0	1.28 × 10^4^	4.29 × 10^2^	1.96 × 10^3^	6.73 × 10^3^	1.12 × 10^4^	1.80 × 10^4^
isorhamnetin 3-*O*-glucoside	C_22_H_22_O_12_	477.1026	8.85	F	1.00 × 10^3^	1.81 × 10^3^	5.92 × 10^3^	1.05 × 10^4^	1.24 × 10^4^	0	0	0	0	0	0	0	1.37 × 10^3^	0	1.33 × 10^4^
tetramethoxyflavone isomer II*	C_19_H_18_O_6_	341.1018	8.67	F	5.12 × 10^2^	0	1.63 × 10^3^	2.27 × 10^3^	2.48 × 10^3^	8.45 × 10^2^	1.43 × 10^3^	3.17 × 10^3^	5.37 × 10^3^	4.84 × 10^3^	0	2.16 × 10^3^	4.39 × 10^3^	0	6.72 × 10^3^
procyanidin trimer C-type II	C_45_H_38_O_18_	865.1956	6.14	F	1.03 × 10^3^	1.57 × 10^3^	8.07 × 10^3^	1.08 × 10^4^	6.29 × 10^3^	0	0	0	0	0	0	0	0	0	0
catechol	C_6_H_6_O_2_	109.0289	0.94	OP	1.29 × 10^3^	2.60 × 10^3^	3.07 × 10^3^	8.17 × 10^3^	1.15 × 10^4^	0	0	0	0	0	0	0	0	0	0
morin	C_15_H_10_O_7_	301.0343	9.80	F	8.09 × 10^2^	1.22 × 10^3^	1.21 × 10^3^	2.93 × 10^3^	3.96 × 10^3^	0	0	0	0	0	0	0	0	0	0
PC in common among all genotypes/growing seasons (*n* = 26)																			
puerarin	C_21_H_20_O_9_	415.1027	9.94	F	0	0	0	0	7.13 × 10^5^	0	0	0	2.76 × 10^6^	2.26 × 10^6^	0	1.18 × 10^3^	0	2.60 × 10^6^	2.64 × 10^6^
4-hydroxybenzaldehyde*	**C**_**7**_**H**_**6**_**O**_**2**_	**121.0289**	**6.14**	**OP**	4.52 × 10^5^	6.97 × 10^5^	1.67 × 10^6^	1.58 × 10^6^	0	4.46 × 10^4^	8.09 × 10^4^	8.80 × 10^4^	7.94 × 10^4^	6.10 × 10^4^	5.35 × 10^4^	4.19 × 10^4^	8.18 × 10^4^	0	2.22 × 10^5^
caffeic acid*	**C**_**9**_**H**_**8**_**O**_**4**_	**179.0343**	**6.24**	**PA**	9.42 × 10^4^	8.05 × 10^4^	1.81 × 10^5^	4.14 × 10^5^	5.29 × 10^5^	1.17 × 10^4^	3.26 × 10^4^	0	4.46 × 10^5^	6.08 × 10^5^	2.02 × 10^4^	4.08 × 10^4^	0	5.10 × 10^5^	3.63 × 10^5^
*trans*-ferulic acid*	**C**_**10**_**H**_**10**_**O**_**4**_	**193.0499**	**8.00**	**PA**	6.70 × 10^4^	1.15 × 10^5^	1.49 × 10^5^	2.28 × 10^5^	1.99 × 10^5^	9.20 × 10^4^	1.92 × 10^5^	2.87 × 10^4^	4.21 × 10^5^	3.43 × 10^5^	4.54 × 10^3^	3.30 × 10^5^	4.53 × 10^5^	3.53 × 10^5^	3.47 × 10^5^
*p*-coumaric acid*	**C**_**9**_**H**_**8**_**O**_**3**_	**163.0393**	**7.48**	**PA**	2.51 × 10^4^	5.07 × 10^4^	1.28 × 10^5^	6.56 × 10^4^	2.38 × 10^5^	6.97 × 10^3^	1.01 × 10^5^	5.60 × 10^4^	2.88 × 10^5^	4.68 × 10^4^	1.03 × 10^4^	1.31 × 10^5^	4.48 × 10^4^	3.20 × 10^5^	4.95 × 10^5^
scutellarein*	C_15_H_10_O_6_	285.0392	10.16	F	8.49 × 10^2^	1.21 × 10^4^	1.66 × 10^4^	0	1.76 × 10^3^	0	2.54 × 10^4^	0	0	2.96 × 10^5^	0	4.65 × 10^4^	1.71 × 10^5^	4.09 × 10^5^	5.54 × 10^5^
3′-hydroxymelanettin*	C_16_H_12_O_6_	299.0551	11.03	F	2.98 × 10^2^	0	4.96 × 10^3^	0	0	5.83 × 10^3^	1.03 × 10^3^	2.45 × 10^3^	0	0	7.92 × 10^3^	3.10 × 10^4^	2.19 × 10^5^	3.35 × 10^5^	6.93 × 10^5^
esculetin*	C_9_H_6_O_4_	177.0185	6.10	OP	2.20 × 10^3^	2.00 × 10^3^	3.73 × 10^3^	1.54 × 10^4^	3.60 × 10^4^	4.56 × 10^3^	1.09 × 10^4^	0	2.50 × 10^5^	3.58 × 10^5^	4.22 × 10^3^	1.09 × 10^4^	4.64 × 10^4^	1.84 × 10^5^	2.72 × 10^5^
ferulic acid*	C_10_H_10_O_4_	193.0498	8.25	PA	3.19 × 10^4^	2.05 × 10^3^	7.31 × 10^3^	9.90 × 10^4^	9.43 × 10^4^	4.36 × 10^4^	3.70 × 10^3^	9.57 × 10^3^	0	1.41 × 10^5^	0	1.65 × 10^5^	2.54 × 10^5^	1.55 × 10^5^	1.68 × 10^5^
naringenin 7-*O*-glucoside*	C_21_H_22_O_10_	433.1128	7.84	F	1.33 × 10^4^	2.52 × 10^4^	5.29 × 10^4^	8.95 × 10^4^	7.92 × 10^4^	0	0	4.75 × 10^4^	0	4.83 × 10^4^	0	3.80 × 10^4^	6.59 × 10^4^	5.78 × 10^4^	6.90 × 10^4^
4-hydroxybenzoic acid*	**C**_**7**_**H**_**6**_**O**_**3**_	**137.0237**	**5.03**	**PA**	1.37 × 10^4^	3.06 × 10^4^	7.30 × 10^4^	1.83 × 10^5^	1.44 × 10^5^	8.51 × 10^3^	4.56 × 10^3^	2.79 × 10^3^	1.12 × 10^4^	1.35 × 10^4^	0	7.17 × 10^3^	4.79 × 10^3^	0	3.59 × 10^4^
dihydroxybenzoic acid isomer I*	C_7_H_6_O_4_	153.0186	3.46	PA	1.70 × 10^4^	3.39 × 10^4^	6.34 × 10^4^	1.78 × 10^5^	2.29 × 10^5^	0	0	0	2.03 × 10^3^	3.72 × 10^3^	5.68 × 10^2^	1.08 × 10^3^	0	2.76 × 10^3^	0
hesperidin*	C_28_H_34_O_15_	609.1881	0.57	F	4.00 × 10^3^	6.16 × 10^3^	1.17 × 10^4^	1.98 × 10^4^	2.36 × 10^4^	4.36 × 10^3^	1.53 × 10^4^	2.16 × 10^4^	2.25 × 10^4^	2.29 × 10^4^	4.74 × 10^3^	1.50 × 10^4^	1.79 × 10^4^	2.11 × 10^4^	2.21 × 10^4^
5-caffeoylquinic acid*	C_16_H_18_O_9_	353.0865	5.87	PA	7.03 × 10^4^	0	2.34 × 10^4^	1.76 × 10^4^	1.44 × 10^4^	0	3.17 × 10^3^	0	8.18 × 10^3^	0	9.82 × 10^3^	1.22 × 10^4^	9.24 × 10^3^	0	2.83 × 10^4^
*p*-anisaldehyde	C_8_H_8_O_2_	135.0444	7.07	OP	3.48 × 10^2^	7.94 × 10^2^	0	0	0	0	0	0	0	8.75 × 10^3^	9.99 × 10^2^	1.35 × 10^4^	0	0	1.63 × 10^5^
3-feruloylquinic acid*	C_17_H_20_O_9_	367.1023	6.07	PA	1.17 × 10^4^	0	1.10 × 10^4^	0	1.26 × 10^4^	1.05 × 10^4^	2.58 × 10^4^	0	1.82 × 10^4^	0	2.72 × 10^4^	2.15 × 10^4^	0	2.64 × 10^4^	1.76 × 10^4^
tetramethoxyflavone isomer III*	C_19_H_18_O_6_	341.1019	10.12	F	2.31 × 10^3^	0	0	0	7.48 × 10^3^	5.03 × 10^3^	1.16 × 10^4^	1.59 × 10^4^	2.00 × 10^4^	1.98 × 10^4^	3.09 × 10^3^	1.14 × 10^4^	1.69 × 10^4^	1.88 × 10^4^	2.86 × 10^4^
naringin 4′-*O*-glucoside*	C_21_H_22_O_10_	433.1128	8.35	F	2.82 × 10^3^	5.73 × 10^3^	1.18 × 10^4^	1.99 × 10^4^	1.58 × 10^4^	0	0	1.13 × 10^4^	1.28 × 10^4^	8.59 × 10^3^	0	8.83 × 10^3^	1.42 × 10^4^	1.20 × 10^4^	1.35 × 10^4^
vanillin*	**C**_**8**_**H**_**8**_**O**_**3**_	**151.0393**	**5.80**	**OP**	2.43 × 10^3^	4.07 × 10^3^	8.14 × 10^3^	1.44 × 10^4^	1.72 × 10^4^	3.05 × 10^3^	0	0	0	7.22 × 10^3^	0	0	0	3.38 × 10^4^	3.86 × 10^4^
tetramethoxyflavone isomer I*	C_19_H_18_O_6_	341.1019	8.30	F	2.08 × 10^3^	3.92 × 10^3^	0	7.93 × 10^3^	7.42 × 10^3^	2.40 × 10^3^	4.57 × 10^3^	0	1.45 × 10^4^	1.31 × 10^4^	0	6.56 × 10^3^	1.12 × 10^4^	1.44 × 10^4^	1.48 × 10^4^
feruloyl glucose*	C_16_H_20_O_9_	355.1023	6.46	PA	9.20 × 10^2^	0	0	0	8.41 × 10^3^	0	6.28 × 10^3^	0	4.16 × 10^3^	0	1.59 × 10^3^	1.26 × 10^4^	2.44 × 10^4^	0	4.42 × 10^3^
luteolin 7-*O*-rutinoside	C_27_H_30_O_15_	593.1497	8.11	F	1.09 × 10^3^	2.56 × 10^3^	0	0	0	0	0	0	6.86 × 10^3^	0	2.01 × 10^3^	0	1.56 × 10^4^	9.81 × 10^3^	0
tetramethoxyflavone isomer II*	C_19_H_18_O_6_	341.1018	8.67	F	5.12 × 10^2^	0	1.63 × 10^3^	2.27 × 10^3^	2.48 × 10^3^	8.45 × 10^2^	1.43 × 10^3^	3.17 × 10^3^	5.37 × 10^3^	4.84 × 10^3^	0	2.16 × 10^3^	4.39 × 10^3^	0	6.72 × 10^3^
procyanidin dimer B-type VII	C_30_H_26_O_12_	577.1334	10.26	F	4.26 × 10^2^	0	0	0	2.20 × 10^3^	0	0	0	0	1.23 × 10^4^	0	1.32 × 10^3^	4.91 × 10^3^	0	0
phenylacetic acid	C_8_H_8_O_2_	135.0444	6.51	PA	3.73 × 10^2^	0	0	0	2.69 × 10^3^	0	0	0	5.41 × 10^3^	3.80 × 10^3^	0	0	0	5.26 × 10^3^	0
procyanidin dimer B-type VI	C_30_H_26_O_12_	577.1341	9.43	F	4.57 × 10^2^	0	0	0	2.33 × 10^3^	0	0	0	0	5.80 × 10^3^	0	9.60 × 10^2^	0	0	0

a *m*/*z* = mass/charge; RT = retention time; F = flavonoids; PA = phenolic acids; OP = other polyphenols. PC in bold represent the reference standards, and PC marked with the “*” symbol are in common in both tables (DAF and genotype/GS). Mean of normalized relative compound abundance is shown for each sample (per grain).

Among the 20 common PC found in sorghum grains, regardless of developmental stage and genotype/GS, seven had previously been reported as the main compounds in mature sorghum: *trans*-ferulic acid, caffeic acid, *p*-coumaric acid, 4-hydroxybenzoic acid, 4-hydroxybenzaldehyde acid, esculetin, and ferulic acid.^21^ Some derivatives of hydroxycinnamic and hydroxybenzoic acids have also been detected, e.g., dimeric hydroxybenzoic acid, ferulic acid glycosylated and esterified with quinic acid, and caffeic acid esterified with quinic acid. In addition to phenolic acids and other polyphenol classes, eight important flavonoids (five aglycones and two glucones) were identified. They are synthesized by the central metabolite of flavonoid biosynthesis (naringenin) and have previously been reported in mature sorghum and other cereals, such as wheat.^21,26,27^

Dimeric and trimeric tannins (procyanidins) were reported among PC independent of the development stage (Table 1, PC in common among all days after flowering). Sorghum is a potential source of condensed tannins that are located in the testa, the structure between the pericarp and the endosperm of the grain.^4^ Tannins are known for their high bioactivity and health benefits,^28^ but its negative impact on sorghum protein digestibility is still considered a problem. In our study, the abundance of these tannins (procyanidins) increases progressively throughout the grain development (respectively, 7- and 6-fold higher between 10DAF and 40DAF). Nonetheless, Table 1 confirms the presence of condensed tannins even in the initial stages of the grain.

In the case of PC in common among all genotypes/GS, it is important to highlight puerarin (Table 1, PC in common among all genotypes/GS) since it was previously reported as one of the main flavonoids in mature sorghum.^21,29^ Synthesized by the isoflavonoid pathway via naringenin, we showed that the synthesis of puerarin occurs mainly at the final stages of grain maturation (33 and 40DAF); it was identified as the most abundant PC in the mature grains of all genotypes/GS.

### 3.4 Evolution of the Phenolic Profile during Sorghum Grain Development

The knowledge of PC biosynthesis in sorghum is essential not only for fundamental research on phenolic characterization but also for improvement of grain quality and health benefits; i.e., the elucidation of phenolic evolution mechanisms can drive the sorghum harvest at the appropriate stages according to the needs. Globally, the number of PC identifications during the sorghum development stages exhibited low variation (between 52 and 69 PC) and irregular behavior (Figure 2A). The highest number of annotations refers to the earliest development stage (10DAF, 69 PC), mainly attributed to the 15 PC exclusively found at this stage. Among them, we found sinapic acid, quercetin, and derivatives of hydroxycinnamic acids (caffeic, ferulic, and *p*-coumaric acids) (identified at level 1, confirmed with phenolic standards).

The relative quantification of the annotated compounds through total ion abundance was evaluated by classes and by genotypes (Figure 2C,D). Phenolic synthesis during grain development was evidenced, mainly between the initial grain developmental stage and mature grains (40DAF was 9-fold higher than 10DAF, based on the cumulative classes). In the initial stages (10 and 17DAF), the class of phenolic acids was more abundant (≅50%), followed by other polyphenols (≅30%) and flavonoids (≅ 20%). In the hard dough stage (25DAF), the abundance distribution of these three phenolic classes is equal (≅33% each). In the mature stages (33 and 40DAF), flavonoids become the predominant class in this cereal (3/5 of total ion abundance). The abundance of lignans was inexpressive throughout the grain development (Supplementary Table 1).

As previously mentioned, PC are derived from the phenylpropanoid biosynthetic pathway, which has as its first step the conversion of phenylalanine and tyrosine to cinnamic and coumaric acid, respectively, by phenylalanine/tyrosine ammonia-lyase.^30^ It can be hypothesized that due to the high presence of free amino acids at the early stages of the grain development,^31^ PC synthesis will be favored throughout its development. The data presented here with sorghum and those previously reported on other developing cereal grains suggest a prevalence of the phenolic acid synthesis in early stages.^17,32,33^ From 25DAF, the flavonoid biosynthesis pathway seems to be prioritized, which indicates the hypothesis of greater action of the enzyme 4-coumarate-CoA ligase. This enzyme converts cinnamic and coumaric acids, respectively, into cinnamoyl-CoA or *p*-coumaroyl-CoA, both precursors of this pathway.

**Figure 3:**
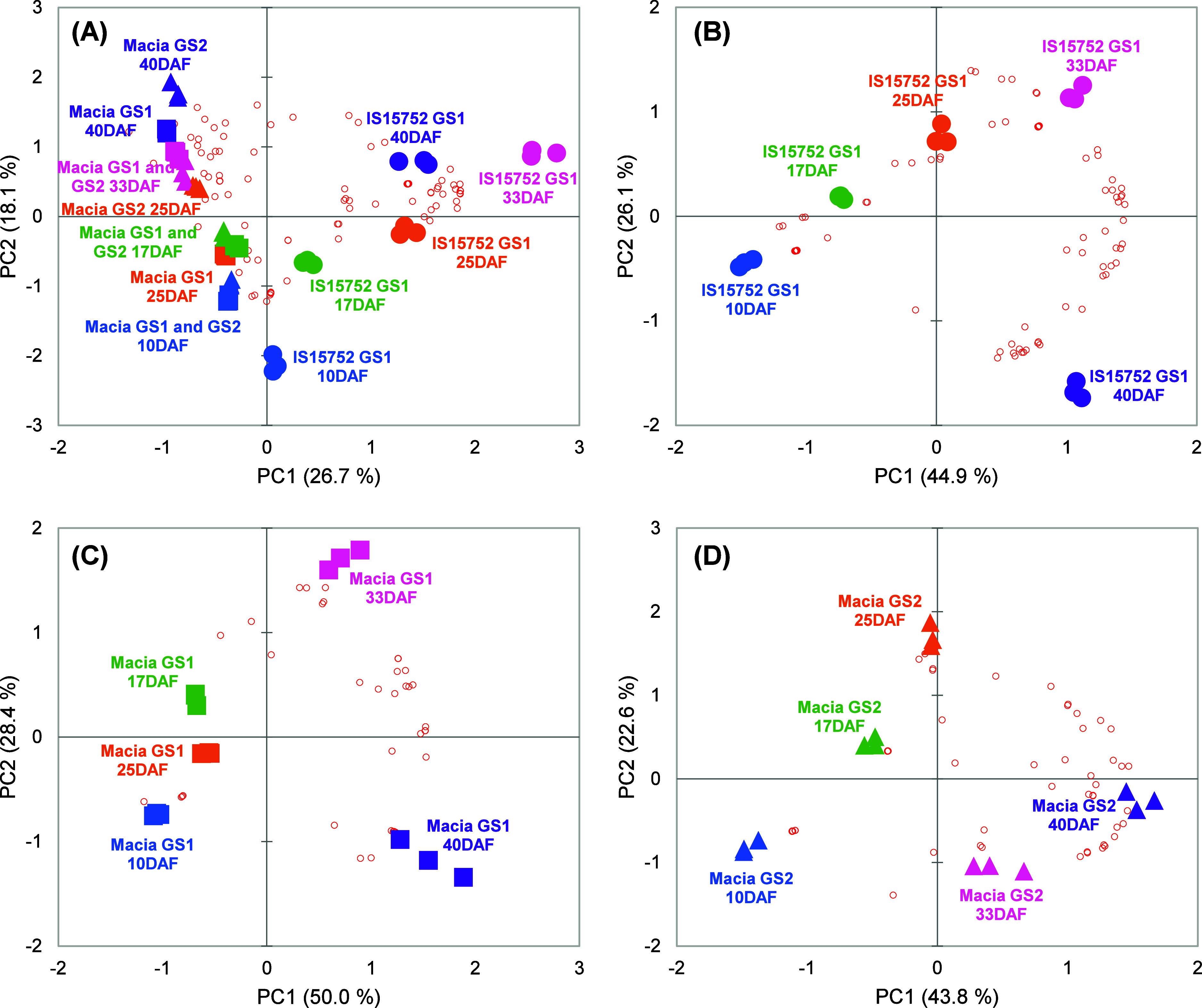
Principal component analysis (PCA) biplot: (A) of all sorghum samples and (B–D) in each genotype/growing season (GS). The samples (symbols) are distributed according to relative intensity of phenolic compounds (red empty circles). (For interpretation of the references to color in this figure legend, the reader is referred to the web version of this article.).

To further explore the variation in the data set, the PCA biplot was applied (Figure 3A). Due to the large number of samples, this first PCA biplot was not efficient to visualize the phenolic variability (PC1 and PC2:45%), but some results were clearly observed: (1) the distinction between genotypes (PC1 27%), where the high-tannin and low-tannin samples were distributed on the right and left side of the *x*-axis, respectively; (2) the separation between the samples of initial stages of grain development (10, 17, and 25DAF on the bottom side) and after physiological maturity (33 and 40DAF on the upper side) (PC2 18%), and (3) the left side of the *x*-axis showed the low influence of GS on phenolic variability.

Each genotype was also evaluated separately, showing a clear distinction between grain development stages (Figure 3B–D). IS15752 GS1 (PC1 and PC2:71%) and Macia GS2 (PC1 and PC2:66%) showed the same behavior, i.e., half-moon distribution of scores, with the earliest stages (10 and 17DAF) located in the left quadrant, mature stages (33 and 40DAF) in the right quadrant, and the intermediate stage (25DAF) centered on the *X*-axis (Figure 3B,D). Macia GS1 showed a similar profile (PC1 and PC2:78%) with a slight difference, and the 25DAF score was grouped together in immature stages (Figure 3C).

**Figure 4:**
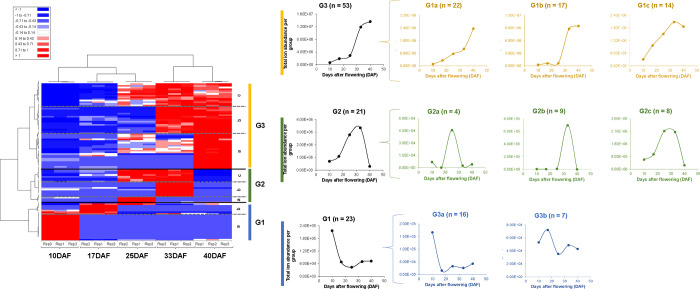
Hierarchical clustering (HCA) heat map of metabolomic data. Three cluster groups (G1–G3) and subclusters were generated using a Pearson correlation (ANOVA, *p* < 0.05) on the differentially abundant phenolic compounds during grain development. Different clusters and subclusters are expressed by the mean of the group total abundance. (For interpretation of the references to color in this figure legend, the reader is referred to the web version of this article).

Hierarchical cluster analysis (HCA) with the correlation matrix (heatmap) was applied with all 97 annotated PC for a better visualization of the different stages and to identify which PC can discriminate each one (Figure 4). First, the vertical HCA separated the samples into two large groups: early stages (10–25DAF) and mature stages (33–40DAF), which corroborates the distribution of scores previously found in the PCA (Figure 3B–D). In the PC characterization, HCA formed three large groups (horizontal axis, G1–G3; Supplementary Table 2) that can be further subdivided (a, b, and c): (1) G1 is represented by 23 PC, more abundant in the early stages of grain development (10–17DAF). Globally, these PC belong to the classes of phenolic acids (52%), followed by flavonoids (30%) and other polyphenols (9%), based on total relative ion abundance (Supplementary Table 2). These phenolic acids are present in G1a and present an abundance reduction at the beginning of the soft dough stage, while G1b presents varied composition and also an abundance reduction in the next stage (25DAF). (2) G2 corresponds to intermediate PC at grain maturation, i.e., an intersection between early stages (represented by 25DAF) and mature stages (represented by 33DAF). This group represents the beginning of the flavonoid synthesis (increase from 30 to 62%), and the reduction of the synthesis of phenolic acids (from 52 to 29%). These alterations in the PC profile can occur at 25DAF (G2a), at 33DAF (G2b), or at both stages (G2c). (3) G3 had the highest number of PC (*n* = 53, Supplementary Table 2) and is represented by the PC synthesized all along grain development, with greater accumulation in mature grains. These compounds are mostly flavonoids (60%), followed by phenolic acids (25%) and other polyphenols (15%). Furthermore, this large group presented three subdivisions, showing that these PC can be progressively synthesized (G3a); synthesized up to 33DAF, with stabilization at 40DAF (G3b); or synthesized up to 33DAF, followed by a reduction in 40DAF (G3c). The HCA multivariate analysis therefore corroborated the data reported in Figure 2B and reinforced the hypothesis of alteration in the route of the phenylpropanoid biosynthetic pathway.

**Figure 5:**
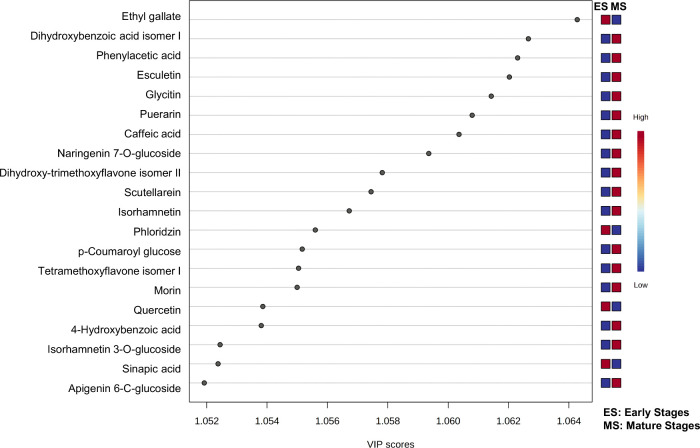
Variable importance in projection (VIP) scores generated from orthogonal partial least-squares discriminant analysis (OPLS-DA). The 20 top important phenolic compounds (VIP score >1.0) contributing to the separation of phenolic profile in early vs mature stages. The relative abundance of phenolic compounds is indicated by a colored scale from blue to red representing the low and high, respectively. (For interpretation of the references to color in this figure legend, the reader is referred to the web version of this article).

Finally, the PC that made important contributions to the classification into two large groups formed by the PCA and HCA (early stages and mature grains) could be selected based on the variable importance in projection (VIP) (Figure 5). According to the online KEGG pathway database (www.genome.jp/kegg/pathway), a schematic diagram of the phenylpropanoid and flavonoid pathway was created with VIP compounds to explain the main degradation/synthesis pathways of these compounds (Supplementary Figure 2). Quercetin, sinapic acid, ethyl gallate, and phloridzin appear as relevant compounds in the early stages of grain development, the first three being specific to the milky stage. Phloridzin is a flavonoid widely reported in plants and has multiple pharmacological effects.^34^ During its biosynthesis, the action of chalcone synthase produces the intermediate compound phloretin, which is converted into phloridzin by glucosylation.^34^ The decrease in this flavonoid in mature sorghum grains may be related to new routes by *p*-coumaroyl-CoA during grain maturation, e.g., the synthesis of *p*-coumaroyl glucose (5-fold higher in mature stages) and/or synthesis of the flavonoids shown in Figure 5 (two-2-fold higher in mature stages).

### 3.5 Growing Season and Genotype Impact on Phenolic Compounds during Sorghum Grain Growth

The number of identifications and the relative quantification by total ion abundance in each GS and/or genotype are shown in Figure 2B,D. As expected, tannin-rich sorghum grains had the highest number of PC, mainly due to its specific PC (54% of the total number of identifications). Among these compounds, several flavonoids have been reported, such as procyanidin dimers and trimers. Macia GS1 and GS2 have 36 PC in common, corroborating the low variability between GS or the low impact of GS on PC accumulation of the same genotype shown by PCA (Figure 3).

The number of identifications showed a strong correlation with the total relative quantification (*r* = 0.84, *p* < 0.05; data not shown). When analyzing the evolution of the relative abundance of these PC during grain growth (Figure 2D), a dissimilar behavior was observed between the variables: (1) Macia GS2 showed a significant and progressive synthesis of PC (40DAF 41-fold higher than 10DAF); (2) IS15752 showed a significant and progressive synthesis up to 33DAF, followed by a decrease (42%) in the final mature stage; (3) Macia GS1 showed the synthesis starting from 25DAF reaching the maximum at 33DAF (15-fold higher than 25DAF).

**Figure 6:**
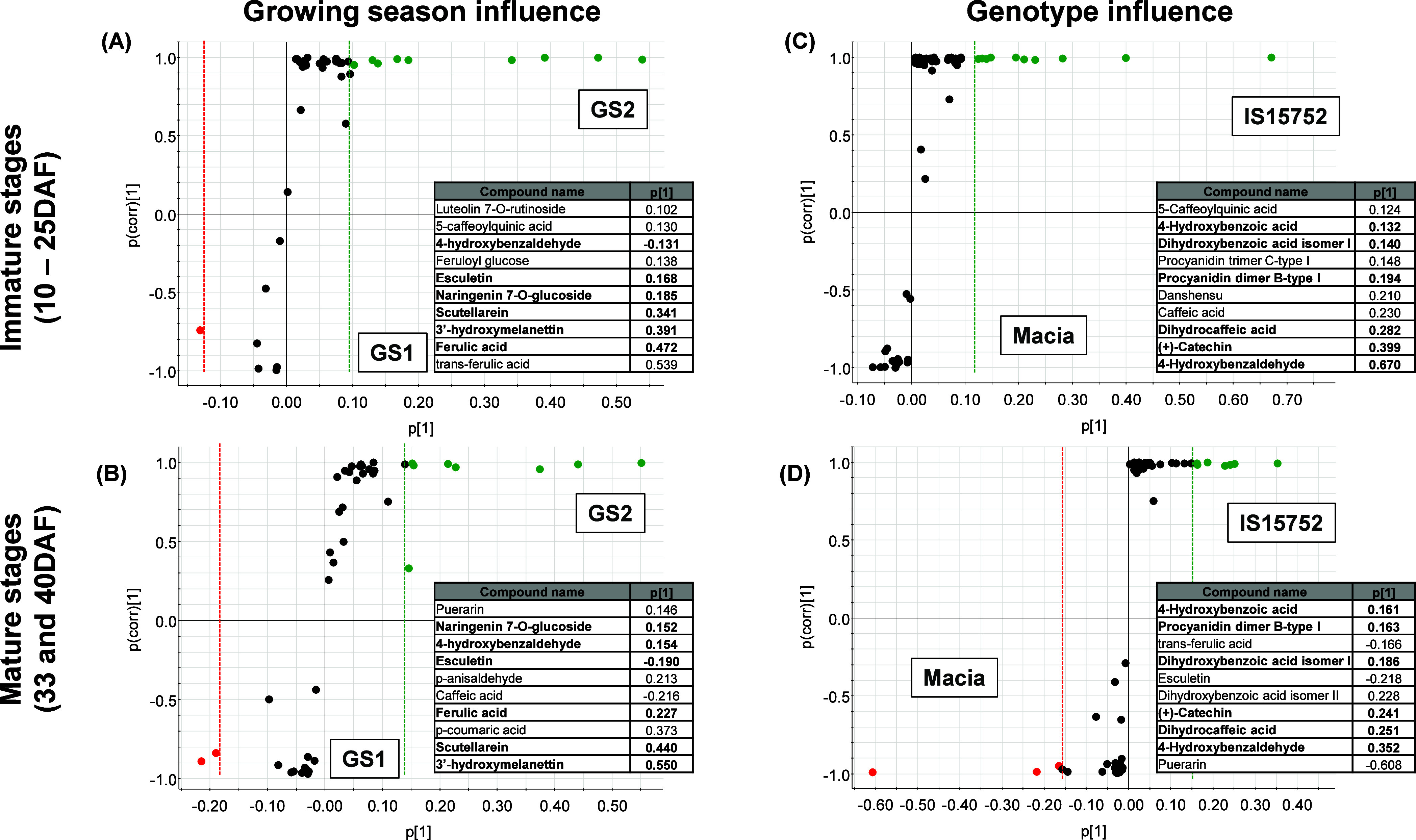
S-plot of orthogonal partial least-squares discriminant analysis (OPLS-DA) between growing season influence (Macia GS1 vs Macia GS2; A, B) and genotype influence (Macia GS1 vs IS15752 GS1; C, D) in developing grains. In the *x*-axis, the relative magnitude of variables (phenolic compounds) is represented, and in the *y* axis, it is the confidence/reliability. Compounds in bold represent phenolics annotated at both immature and mature stages for each treatment (GS or genotype). Variables farthest from the origin in the plot have higher covariance (*p*[1]) and deemed significant markers. Inset tables show the phenolic compound name in ascending order of covariance. (For interpretation of the references to color in this figure legend, the reader is referred to the web version of this article).

The OPLS-DA was also applied in the phenolic profile between GS and genotypes (Figure 6). The OPLS-DA model parameters were robust in early stages (GS: R2Y = 0.998, Q2 = 0.987; genotype: R2Y = 1.000, Q2 = 0.998) and mature stages (GS: R2Y = 0.998, Q2 = 0.977; genotype: R2Y = 1.00, Q2 = 0.996) samples. A total of 10 PC in each S-plot were selected based on the VIP and *p*-value of the OPLS-DA model. From these selected PC in GS and genotype variables, six were found simultaneously on early and mature stages (inset table, Figure 6): 4-hydroxybenzaldehyde, esculetin, naringenin 7-*O*-glucoside, scutellarein, 3′-hydroxymelanettin, and ferulic acid for the GS influence; and 4-hydroxybenzoic acid, dihydroxybenzoic acid isomer I, procyanidin dimer B-type I, dihydrocaffeic acid, (+)-catechin, and 4-hydroxybenzaldehyde for the genotype influence.

Quantitative and qualitative occurrence of plant PC can be associated with several agronomical important phenotypic, i.e., the phenolic diversity between the same genotype of a grain cultivated in different GS may indicate possible biotic and abiotic stresses.^35^ Evaluating the GS influence (Figure 6A,B), four PC have similar behavior, regardless of the development stage analyzed (early or mature stages). These compounds were characteristic of GS2 (green circles) (Figure 5A): [M–H]^−^ 7.84 *m*/*z* 433.1128, [M–H]^−^ 8.25 *m*/*z* 193.0498, [M–H]^−^ 10.16 *m*/*z* 285.0392, and [M–H]^−^ 11.03 *m*/*z* 299.0551, respectively, identified as naringenin 7-*O*-glucoside, ferulic acid, scutellarein, and 3′-hydroxymelanettin. Šamec et al.^36^ emphasized the heightened susceptibility of the flavanone subclass (e.g., naringenin 7-*O*-glucoside) to heat stress, attributing this vulnerability to the presence of two hydroxyl groups in its B ring. Ferulic acid has also previously been pointed as a differentially abundant compound among GS, as its increase is inversely proportional to drought stress.^37^ Although sorghum is known as a tolerant crop, the hypothesis is that Macia GS1 experienced abiotic stress during its cultivation and that was enough to change the phenolic profile of this sample.^36^

Finally, regarding the comparison between different genotypes of the same GS, phenolic acids were the major discriminant metabolites (57%), followed by flavonoids (36%) and other polyphenols (7%) (Figure 6C,D). As expected, the presence of flavanols (characteristic of the tannin-rich genotype IS15752 GS1, green circles) was crucial for the differentiation between genotypes. Among them, we can mention monomers and oligomers of tannins such as catechin—the most commonly monomer reported in sorghum grains—and dimers and trimers of tannins. Although the literature mostly associates these compounds with antinutritional factors, tannins have high health-promoting ability and their dimers are well absorbed by the human body.^5^ They also appear to play an important role in the food industry, particularly as high-value ingredients to naturally modify and expand protein functionality.^38,39^ Evidence indicates that polymeric sorghum tannins can drastically alter the rheological behavior of gluten in blended flours, being able to increase gluten-force.^38^

In conclusion, this is the most up-to-date work to show a comprehensive study of the synthesis of phenolic compounds in developing sorghum grain. The phenolic content increased during grain filling, and the flavonoid biosynthesis pathway seems to be prioritized from 25DAF, potentially explaining the higher antioxidant activity in mature grains. The metabolomic approach also revealed the presence of hydroxycinnamic and hydroxybenzoic acids, as well as their derivatives, at all grain stages, except for lignans that were not identified in mature grains. Chemometric analysis showed discriminatory compounds among stages, genotypes, and growing seasons. Genotypes had more impact on phenolic profile variability, mainly due to the high presence of condensed tannins, while growing season seems to less influence the polyphenol content but has important biomarkers in this differentiation, e.g., 4-hydroxybenzaldehyde. Monomers, dimers, and trimers of procyanidins specific to the tannin-rich genotype were also annotated in the tannin-free genotype. Our results revealed the complex development of phenolic compounds in sorghum grains, which can contribute to open polyphenol databases and encourage greater exploitation by the agrifood industry to obtain health-promoting grains by selecting genotypes and developing stages with an optimized composition in bioactive compounds.

## Supporting Information Available

Photographs of each sorghum genotype and corresponding average grain weight (Supplementary Figure 1); schematic diagram of the phenylpropanoid and flavonoids pathway associated with the synthesis and degradation of phenolic compounds during days after flowering (Supplementary Figure 2); all phenolic compounds tentatively identified by UHPLC-MS^E^ in retention time order (Supplementary Table 1); list of phenolic compounds grouped according to the hierarchical cluster analysis (HCA) presented in Figure 4 (Supplementary Table 2) (PDF)

## Abbreviations and nomenclature

BPC: bound phenolic compound
DAF: days after flowering
DM: dry mass
DPPH: 2,2-diphenyl-1-picrylhydrazyl
ESI: electrospray ionization
FPC: free phenolic compound
FRAP: ferric-reducing antioxidant power
GAE: gallic acid equivalent
GS: growing season
HCA: hierarchical cluster analysis
KEGG: Kyoto Encyclopedia of Genes and Genomes
MS: mass spectrometer
OPLS-DA: orthogonal partial least-squares discriminant analysis
PC: phenolic compound
PCA: principal component analysis
PTFE: polytetrafluoroethylene
QC: quality control
QTOF: quadrupole time-of-flight
TE: trolox equivalent
TPC: total phenolic compounds
TPTZ: 2,4,6-tri(2-piridil)1-3-5-triazine
TRC: total reducing capacity
UHPLC: ultrahigh-performance liquid chromatography
VIP: variable importance in projection
