# Supplementary material for: Dynamic Metabolomic Changes in the Phenolic Compound Profile and Antioxidant Activity in Developmental Sorghum Grains
Source: J Agric Food Chem. 2024 Dec 30;73(2):1725–38. doi: 10.1021/acs.jafc.4c08975 (PMC11741113; doi:10.1021/acs.jafc.4c08975)
Supplement: Supplementary file 1 — jf4c08975_si_001.pdf [file jf4c08975_si_001.pdf]

Supporting Information

Dynamic metabolomic changes in the phenolic compounds profile and antioxidant activity in developmental sorghum grains

Carolina THOMAZ DOS SANTOS D'ALMEIDA[1,2], Marie-Hélène MOREL[3], Nancy TERRIER[4], Hamza MAMERI[3], Mariana SIMÕES LARRAZ FERREIRA[1,2]*

[1] Laboratory of Bioactives (LABBIO), Food and Nutrition Graduate Program (PPGAN), Federal University of the State of Rio de Janeiro (UNIRIO), Rio de Janeiro, 22290-240, Brazil.
[2] Center of Innovation in Mass Spectrometry, Laboratory of Protein Biochemistry, UNIRIO, Rio de Janeiro, 22290-240, Brazil.
[3] UMR 1208 IATE, Univ Montpellier, INRAE, L'Institut-Agro Montpellier, F-34060, France
[4] AGAP Institute, Univ. Montpellier, INRAE, CIRAD, F-34398 Montpellier, France

* mariana.ferreira@unirio.br

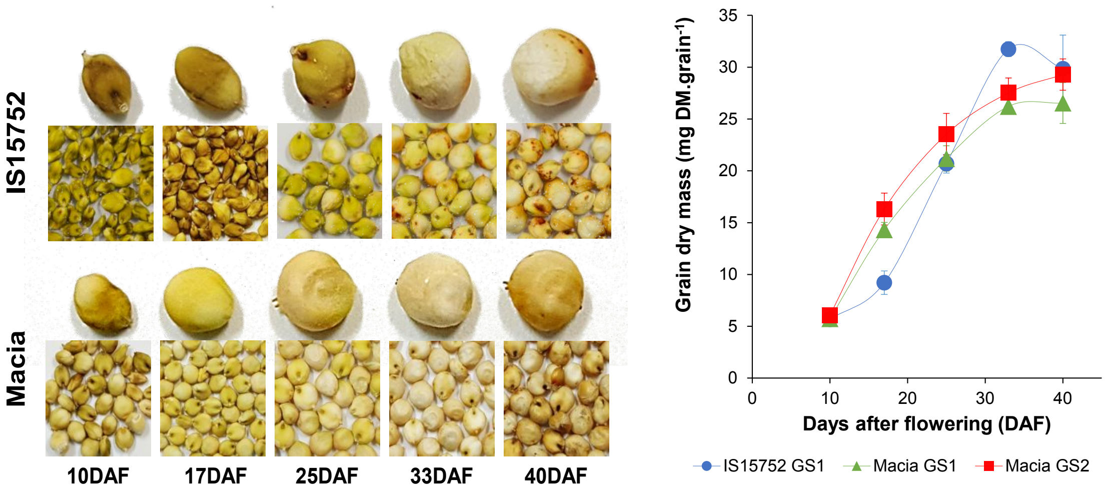

Supplementary Figure 1. Photograph of each genotype (on the left) and the average weight (on the right) on each day after flowering (DAF) of the sorghum grain. Results are expressed as mean ± standard deviation (n=3).

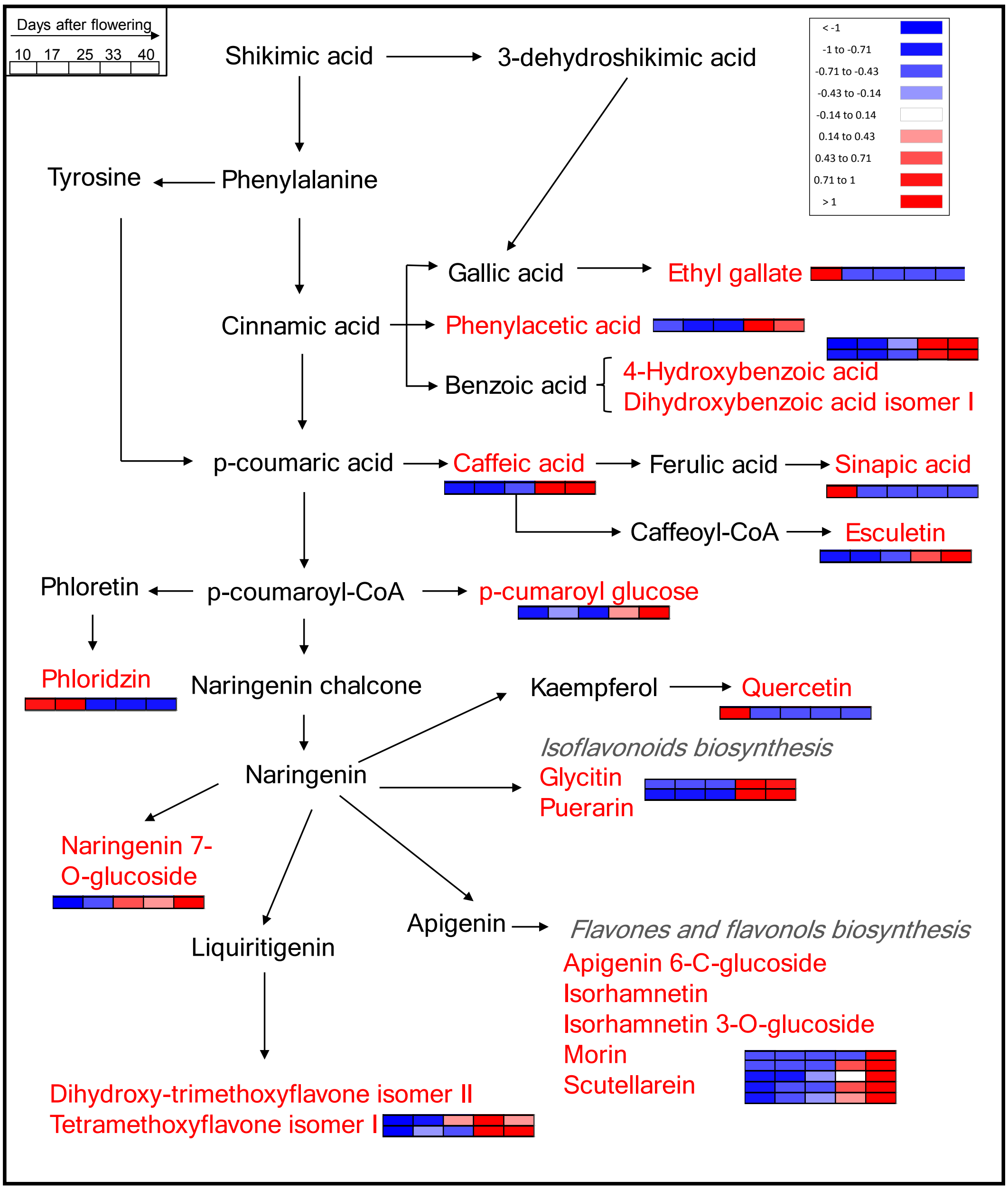

Supplementary Figure 2. Schematic diagram of phenylpropanoid and flavonoids pathway associated with the synthesis and degradation of phenolic compounds during days after flowering (DAF). The compounds in red were selected by the Variable Importance in Prediction (VIP, see Supplementary Figure 3). The small heat maps represents normalized relative metabolite abundance of grains at a different stage from 10 to 40DAF, respectively. Red color represents higher abundance, and blue color represents lower abundance.

Supplementary Table 1. All phenolic compounds tentatively identified by UHPLC-MS^{E} in retention time order.

Name of compound	Molecular formula	*m/z*	RT (min)	Score (%)	FS (%)	Error (ppm)	IS (%)	Class	IS15752 GS1										Macia GS1										Macia GS2									
									10DAF F	10DAF B	17DAF F	17DAF B	25DAF F	25DAF B	33DAF F	33DAF B	40DAF F	40DAF B	10DAF F	10DAF B	17DAF F	17DAF B	25DAF F	25DAF B	33DAF F	33DAF B	40DAF F	40DAF B	10DAF F	10DAF B	17DAF F	17DAF B	25DAF F	25DAF B	33DAF F	33DAF B	40DAF F	40DAF B
Dihydroformononetin	C16H14O4	269.0835	0.55	36.5	0.5	5.83	88.73	F					1.26E+03		1.95E+03																							
	C28H34O15	609.1881	0.57	44.9	44.2	9.22	90.57	F	4.00E+03		6.16E+03		1.17E+04		1.98E+04		2.36E+04		4.36E+03		1.53E+04		2.16E+04		2.25E+04		2.29E+04		4.74E+03		1.50E+04		1.79E+04		2.11E+04		2.21E+04	
Nortrachelogenin/7-hydroxymatairesinol/isohydroxymatairesinol	C20H22O7	373.1299	0.60	37.8	0.6	1.79	90.54	L	3.52E+02										4.12E+02																			
Catechol	C6H6O2	109.0289	0.94	54.4	83.8	-5.80	94.76	OP		1.29E+03		2.60E+03		3.07E+03		8.17E+03		1.15E+04																				
Ethyl gallate	C9H10O5	197.0445	2.10	36.7	0.0	-5.15	89.58	PA		2.94E+02																												
Danshensu	C9H10O5	197.0448	3.10	55.9	84.4	-3.74	99.51	PA		3.33E+04		5.94E+04		1.66E+05		2.53E+05																						
Dihydroxybenzoic acid isomer I	C7H6O4	153.0186	3.46	57.1	91.7	-4.81	99.52	PA	3.83E+03	1.31E+04	5.89E+03	2.81E+04	1.14E+04	5.21E+04	2.11E+04	1.57E+05	3.19E+04	1.97E+05								2.03E+03		3.72E+03	5.68E+02		1.08E+03					2.76E+03		
3,4-dihydroxyphenylacetic acid	C8H8O4	167.0343	3.80	57.3	96.1	-4.04	95.16	PA				5.32E+03		7.34E+03		1.18E+04		1.25E+04																				
Caffeoylquinic acid isomer I	C16H18O9	353.0868	4.14	57.3	98.1	-2.95	91.96	PA	1.98E+03																				4.17E+02									
Methylcatechol isomer	C7H8O2	123.0445	4.21	37.9	0.0	-5.56	95.70	OP				2.14E+03		2.29E+03		3.69E+03		3.19E+03																				
3,4-dihydroxyphenylglycol	C8H10O4	169.0497	4.21	37.3	0.0	-5.34	92.49	OP				8.84E+02																										
Vanillactic acid	C10H12O5	211.0608	4.25	45.4	35.1	-1.67	93.73	PA																			2.95E+03										1.60E+03	
Dihydrocaffeic acid	C9H10O4	181.0500	4.46	56.5	86.8	-3.31	99.46	PA		6.56E+04		1.23E+05		2.74E+05		3.84E+05		3.47E+05																				
Homovanillic acid	C9H10O4	181.0499	4.73	54.8	80.2	-4.18	98.87	PA		1.36E+04		2.59E+04				7.82E+04		6.69E+04																				
**4-hydroxybenzoic acid**	**C7H6O3**	**137.0237**	**5.03**	**55.9**	**85.9**	**-4.97**	**99.08**	**PA**	**8.38E+03**	**5.31E+03**	**2.12E+04**	**9.42E+03**	**5.56E+04**	**1.74E+04**	**1.48E+05**	**3.49E+04**	**1.03E+05**	**4.14E+04**	**6.89E+03**	**1.62E+03**	**4.56E+03**			**2.79E+03**		**1.12E+04**	**1.35E+04**				**7.17E+03**		**4.79E+03**				**1.01E+04**	**2.58E+04**
**2,5-dihydroxybenzoic acid**	**C7H6O4**	**153.0186**	**5.05**	**46.9**	**40.3**	**-4.48**	**99.55**	**PA**	**4.12E+02**	**4.89E+04**																												
Procyanidin dimer B-type I	C30H26O12	577.1338	5.42	55.3	80.9	-2.32	98.24	F	2.12E+04		3.50E+04		1.63E+05		1.86E+05		1.25E+05																					
Caffeoylquinic acid isomer II	C16H18O9	353.0867	5.46	37.5	0.0	-3.22	91.18	PA	8.34E+02																													
Paeonol	C9H10O3	165.0551	5.55	41.8	17.3	-3.98	96.31	OP				1.12E+03																										
Ferulaldehyde	C9H6O4	177.0185	5.58	38.0	0.0	-4.65	95.16	OP		1.34E+03		2.08E+03		6.58E+03				1.37E+04																				
Procyanidin trimer C-type I	C45H38O18	865.1963	5.76	56.0	91.4	-2.64	91.77	F	1.23E+04		2.04E+04		9.39E+04		1.11E+05		9.14E+04																					
**Vanillin**	**C8H8O3**	**151.0393**	**5.80**	**53.1**	**73.3**	**-4.92**	**97.76**	**OP**		**2.43E+03**		**4.07E+03**		**8.14E+03**		**1.44E+04**		**1.72E+04**		**3.05E+03**							**7.22E+03**									**3.38E+04**		**3.86E+04**
p-coumaric acid 4-O-glucoside	C15H18O8	325.0915	5.82	54.5	92.8	-4.35	85.03	PA											3.00E+02										3.25E+02									
5-caffeoylquinic acid	C16H18O9	353.0865	5.87	57.7	94.0	-3.73	98.86	PA	7.03E+04				2.34E+04		1.76E+04		1.44E+04				3.17E+03				8.18E+03				9.82E+03		1.22E+04		9.24E+03				2.83E+04	
**(+)-catechin**	**C15H14O6**	**289.0710**	**5.91**	**47.9**	**43.9**	**-2.70**	**98.63**	**F**	**9.35E+04**		**1.68E+05**		**6.62E+05**		**6.78E+05**																							
p-coumaroyl glucose	C15H18O8	325.0910	5.96	42.5	29.2	-5.91	90.03	PA							7.11E+03		8.79E+03												7.78E+02		3.48E+03						6.73E+03	
Methoxyphenylacetic acid	C9H10O3	165.0550	6.00	38.1	0.0	-4.58	95.62	PA	2.86E+02			3.06E+03						8.34E+03																				
3-feruloylquinic acid	C17H20O9	367.1023	6.07	56.9	92.4	-3.16	95.84	PA	1.17E+04				1.10E+04				1.26E+04		1.05E+04		2.58E+04				1.82E+04				2.72E+04		2.15E+04				2.64E+04		1.76E+04	
Esculetin	C9H6O4	177.0185	6.10	54.5	79.1	-4.43	98.58	OP	2.20E+03		2.00E+03		3.73E+03		1.54E+04		3.60E+04		4.56E+03		1.09E+04				2.50E+05		3.58E+05		4.22E+03		1.09E+04		4.64E+04		1.84E+05		2.72E+05	
**4-hydroxybenzaldehyde**	**C7H6O2**	**121.0289**	**6.14**	**56.6**	**90.2**	**-5.22**	**98.83**	**OP**	**4.52E+05**		**6.97E+05**		**1.67E+06**		**1.58E+06**				**1.50E+04**	**2.96E+04**	**5.27E+04**	**2.83E+04**	**8.80E+04**			**7.94E+04**	**6.10E+04**		**3.96E+04**	**1.39E+04**		**4.19E+04**		**8.18E+04**			**5.53E+04**	**1.67E+05**
4-hydroxymandelic acid	C8H8O4	167.0342	6.14	56.4	90.4	-4.63	96.96	PA	7.98E+03		1.18E+04		2.95E+04		2.91E+04																							
Procyanidin trimer C-type II	C45H38O18	865.1956	6.14	54.4	92.0	-3.44	83.95	F	1.03E+03		1.57E+03		8.07E+03		1.08E+04		6.29E+03																					
**Caffeic acid**	**C9H8O4**	**179.0343**	**6.24**	**56.7**	**89.6**	**-4.01**	**98.69**	**PA**	**2.58E+04**	**6.84E+04**	**1.84E+04**	**6.21E+04**	**9.03E+04**	**9.10E+04**	**2.64E+05**	**1.50E+05**	**3.54E+05**	**1.75E+05**	**1.17E+04**		**3.26E+04**				**4.46E+05**		**3.71E+05**	**2.37E+05**	**2.02E+04**		**4.08E+04**				**3.49E+05**	**1.62E+05**	**3.63E+05**	
Dihydroquercetin	C15H12O7	303.0505	6.34	51.9	75.1	-1.87	86.59	F					1.31E+03																									
Feruloyl glucose	C16H20O9	355.1023	6.46	53.1	77.9	-3.13	91.42	PA	9.20E+02								8.41E+03				6.28E+03				4.16E+03				1.59E+03		1.26E+04		2.44E+04				4.42E+03	
Phenylacetic acid	C8H8O2	135.0444	6.51	38.1	0.0	-5.56	96.89	PA		3.73E+02							2.69E+03								5.41E+03		3.80E+03								5.26E+03			
Umbelliferone	C9H6O3	161.0236	6.60	54.0	84.3	-4.85	91.12	OP																	3.34E+03		1.97E+03								2.78E+03		3.07E+03	
Caffeoylquinic acid isomer III	C16H18O9	353.0863	6.64	52.6	69.2	-4.13	98.43	PA	2.71E+04																				1.04E+03									
4-p-coumaroylquinic acid	C16H18O8	337.0917	6.85	42.7	24.9	-3.47	92.41	PA	1.85E+03																								1.19E+03		7.42E+03			
Eriodictyol 7-O-glucoside	C21H22O11	449.1073	6.95	56.6	92.9	-3.56	94.29	F	2.65E+03		5.41E+03		2.07E+04		3.57E+04		2.96E+04																					
Feruloylquinic acid isomer	C17H20O9	367.1024	6.96	39.0	8.5	-2.85	89.69	PA	4.18E+02																				6.46E+02									
Dihydroxybenzoic acid isomer II	C7H6O4	153.0187	7.05	41.7	14.1	-4.28	99.33	PA	9.52E+02		1.69E+03		5.95E+03		8.03E+03	6.06E+05																						
2,3,4-trihydroxybenzoic acid	C7H6O5	169.0134	7.05	55.0	87.2	-4.83	93.28	PA								9.59E+03																						
p-anisaldehyde	C8H8O2	135.0444	7.07	57.1	92.4	-5.22	98.99	OP		3.48E+02		7.94E+02															8.75E+03			9.99E+02		1.35E+04						1.63E+05
Cinnamoyl glucose	C15H18O7	309.0998	7.12	37.6	10.9	5.74	83.51	PA					1.33E+03		1.97E+03																							
**4-hydroxyphenylacetic acid**	**C8H8O3**	**151.0393**	**7.16**	**38.1**	**0.0**	**-4.94**	**96.01**	**PA**												**3.71E+02**																		
Dihydroquercetin 3-O-rhamnoside	C21H22O11	449.1080	7.32	35.0	0.0	-2.10	77.54	F																							1.13E+03							
**p-coumaric acid**	**C9H8O3**	**163.0393**	**7.48**	**54.8**	**80.7**	**-4.47**	**98.66**	**PA**	**4.33E+03**	**2.08E+04**		**5.07E+04**	**3.78E+04**	**8.99E+04**	**6.56E+04**		**7.37E+04**	**1.64E+05**	**6.97E+03**		**1.59E+04**	**8.52E+04**	**5.60E+04**		**7.08E+04**	**2.17E+05**	**4.68E+04**		**1.03E+04**		**1.82E+04**	**1.13E+05**	**4.48E+04**		**7.23E+04**	**2.47E+05**	**7.34E+04**	**4.22E+05**
Procyanidin dimer B-type III	C30H26O12	577.1335	7.48	53.4	75.7	-2.92	94.52	F	4.13E+03		6.10E+03		2.15E+04		2.02E+04		1.55E+04																					
Eriodictyol 7-O-glucoside	C21H22O11	449.1081	7.50	54.0	85.5	-1.85	86.81	F					2.13E+03																									
Naringenin 7-O-glucoside	C21H22O10	433.1128	7.84	50.2	55.5	-2.86	98.92	F	1.21E+03	1.21E+04		2.52E+04		5.29E+04		8.95E+04		7.92E+04						4.75E+04				4.83E+04				3.80E+04		6.59E+04		5.78E+04		6.90E+04
**trans-ferulic acid**	**C10H10O4**	**193.0499**	**8.00**	**42.0**	**16.5**	**-3.95**	**98.12**	**PA**	**2.33E+03**	**6.47E+04**	**5.15E+03**	**1.09E+05**	**1.88E+04**	**1.30E+05**	**2.68E+04**	**2.01E+05**	**2.08E+04**	**1.78E+05**	**3.81E+03**	**8.81E+04**	**1.05E+04**	**1.81E+05**	**2.87E+04**		**2.46E+04**	**3.96E+05**		**3.43E+05**	**4.54E+03**		**1.10E+04**	**3.19E+05**	**1.59E+04**	**4.37E+05**	**1.25E+04**	**3.41E+05**	**8.73E+03**	**3.38E+05**
Eriodictyol	C15H12O6	287.0557	8.00	42.3	26.7	-1.44	86.36	F		5.28E+02																												
**Quercetin**	**C15H10O7**	**301.0347**	**8.01**	**43.5**	**33.7**	**-2.37**	**86.39**	**F**		**3.73E+02**																												
Quercetin 3-O-rutinoside	C27H30O16	609.1447	8.07	48.3	51.4	-2.33	92.86	F	5.30E+03		7.21E+03		1.50E+04		1.48E+04		1.45E+04																					
**Sinapic acid**	**C11H12O5**	**223.0602**	**8.09**	**56.5**	**92.1**	**-4.23**	**95.20**	**PA**																					**3.15E+02**									
Luteolin 7-O-rutinoside	C27H30O15	593.1497	8.11	45.8	44.3	-2.50	87.63	F	1.09E+03		2.56E+03														6.86E+03				2.01E+03				1.56E+04		9.81E+03			
Ferulic acid	C10H10O4	193.0498	8.25	42.9	21.1	-4.30	98.58	PA	9.61E+02	3.10E+04	2.05E+03		7.31E+03			9.90E+04	8.52E+03	8.58E+04	1.32E+03	4.22E+04	3.70E+03		9.57E+03					1.41E+05			3.84E+03	1.62E+05	1.13E+04	2.42E+05	9.51E+03	1.45E+05	1.26E+04	1.55E+05
Tetramethoxyflavone isomer I	C19H18O6	341.1019	8.30	42.6	21.3	-3.40	95.75	F		2.08E+03		3.92E+03				7.93E+03		7.42E+03		2.40E+03		4.57E+03				1.45E+04		1.31E+04				6.56E+03		1.12E+04		1.44E+04		1.48E+04
Flavonoid derivative compound	C33H40O21	771.1914	8.30	43.9	37.4	-9.81	92.81	F				1.12E+03				1.83E+03																1.37E+03		3.31E+03				4.26E+03
6-hydroxyluteolin	C15H10O7	301.0343	8.35	40.3	16.8	-3.50	88.94	F		1.26E+03								8.54E+03																				
Naringin 4'-O-glucoside	C21H22O10	433.1128	8.35	44.3	31.2	-2.84	93.59	F		2.82E+03		5.73E+03		1.18E+04		1.99E+04		1.58E+04						1.13E+04		1.28E+04		8.59E+03				8.83E+03		1.42E+04		1.20E+04		1.35E+04
Glycitein	C16H12O5	283.0601	8.43	38.6	7.0	-3.94	90.35	F							3.56E+03																							
Chrysoeriol 7-O-apiosyl-glucoside	C27H30O15	593.1497	8.53	48.6	56.9	-2.44	89.17	F	4.26E+02		7.86E+02						2.67E+03																					
Phloridzin	C21H24O10	435.1281	8.55	37.8	2.9	-3.52	90.04	F	5.01E+02		6.98E+02																											
Isorhamnetin 3-O-rutinoside	C22H22O11	461.1071	8.59	36.8	0.6	-3.88	87.70	F	5.53E+02		7.37E+02																				1.46E+03							
Tetramethoxyflavone isomer II	C19H18O6	341.1018	8.67	39.0	9.0	-3.77	90.18	F		5.12E+02				1.63E+03		2.27E+03		2.48E+03		8.45E+02		1.43E+03		3.17E+03		5.37E+03		4.84E+03				2.16E+03		4.39E+03				6.72E+03
Neoeriocitrin	C27H32O15	595.1658	8.69	41.2	23.1	-1.73	85.17	F																					4.39E+02									
Isorhamnetin 3-O-glucoside	C22H22O12	477.1026	8.85	45.6	40.1	-2.60	90.88	F	1.00E+03		1.81E+03		5.92E+03		1.05E+04		1.24E+04																1.37E+03				1.33E+04	
Apigenin 6-C-glucoside	C21H20O10	431.0976	8.87	48.4	56.2	-1.77	88.09	F	3.37E+01																												1.31E+04	
Hesperetin	C16H14O6	301.0710	9.01	56.0	84.2	-2.57	98.73	F		6.47E+03				3.31E+04																								
Procyanidin dimer B-type V	C27H30O14	577.1548	9.01	48.9	59.7	-2.61	88.19	F										7.69E+01																	2.67E+04			
Tectoridin	C22H22O11	461.1072	9.04	40.3	20.5	-3.70	85.42	F																			3.36E+03											
Dihydroxy-trimethoxyflavone isomer I	C18H16O7	343.0811	9.05	49.4	62.2	-3.52	88.93	F																		4.66E+03		6.18E+03				1.78E+03		3.28E+03				6.39E+03
Procyanidin dimer B-type VI	C30H26O12	577.1341	9.43	49.1	67.0	-1.80	80.78	F		4.57E+02								2.33E+03														9.60E+02						
Rhamnetin	C16H12O7	315.0500	9.55	39.9	16.8	-3.38	86.59	F		8.55E+02								6.46E+03																				
Trihydroxyisoflavanone isomer	C15H12O5	271.0600	9.59	42.5	26.8	-4.22	90.53	F		4.38E+02																												
Homoeriodictyol	C16H14O6	301.0702	9.64	40.0	16.0	-5.15	90.15	F							6.27E+03																							
Morin	C15H10O7	301.0343	9.80	48.5	56.7	-3.59	89.94	F		8.09E+02		1.22E+03		1.21E+03		2.93E+03		3.96E+03																				
Sakuranetin	C16H14O5	285.0757	9.89	37.4	2.0	-3.86	89.44	F							1.08E+04																							
Dihydroxy-trimethoxyflavone isomer II	C18H16O7	343.0811	9.93	43.1	23.4	-3.43	95.95	F												2.11E+03		3.70E+03		8.89E+03		2.08E+04		2.61E+04		1.61E+03		6.37E+03		1.60E+04		2.19E+04		
Puerarin	C21H20O9	415.1027	9.94	57.1	88.8	-1.71	98.91	F									7.13E+05								2.76E+06		2.25E+06	8.69E+03				1.18E+03			2.60E+06		2.63E+06	1.34E+04
Tetramethoxyflavone isomer III	C19H18O6	341.1019	10.12	43.5	25.1	-3.48	96.65	F		2.31E+03								7.48E+03		5.03E+03		1.16E+04		1.59E+04		2.00E+04		1.98E+04		3.09E+03		1.14E+04		1.69E+04		1.88E+04		2.86E+04
Unknown compound	C10H8O4	191.0343	10.16	40.3	12.6	-3.60	93.01	NI		5.45E+02																												
Scutellarein	C15H10O6	285.0392	10.16	46.8	42.2	-4.37	97.11	F		8.49E+02	1.21E+04		1.66E+04					1.76E+03			2.54E+04						2.80E+05	1.57E+04			4.65E+04		1.71E+05		4.09E+05		5.54E+05	
Procyanidin dimer B-type VII	C30H26O12	577.1334	10.26	38.4	10.4	-2.94	85.03	F		4.26E+02								2.20E+03										1.23E+04				1.32E+03		4.91E+03				
Coumestrol	C15H8O5	267.0289	10.36	51.4	69.6	-3.63	91.80	OP									2.24E+03																					
1-Acetoxypinoresinol	C22H24O8	415.1394	10.50	40.5	12.2	-1.18	91.56	L														4.78E+03				1.08E+04								9.91E+03				
Glycitin	C22H22O10	445.1126	10.55	52.5	72.3	-3.14	93.81	F																	1.55E+05		9.13E+04								1.68E+05		1.30E+05	
Dihydroxy-trimethoxyflavone isomer III	C18H16O7	343.0812	10.59	46.4	42.7	-3.15	93.10	F																				1.28E+04		4.29E+02		1.96E+03		6.73E+03		1.12E+04		1.80E+04
Procyanidin dimer B-type VIII	C30H26O12	577.1333	10.71	36.4	4.4	-3.26	81.66	F																				3.29E+03						1.34E+03				
Isorhamnetin	C16H12O7	315.0501	10.92	48.3	57.8	-2.87	86.99	F																											9.47E+03		1.47E+04	
Jaceosidin	C17H14O7	329.0655	10.96	47.4	44.8	-3.48	96.12	F	9.53E+02		1.47E+03				6.28E+03																							
3'-hydroxymelanettin	C16H12O6	299.0551	11.03	53.6	74.6	-3.25	97.00	F		2.98E+02			4.96E+03						5.83E+03		1.03E+03		2.45E+03						7.14E+03	7.83E+02	2.83E+04	2.71E+03	2.08E+05	1.07E+04	3.35E+05		6.93E+05	
Nepetin	C16H12O7	315.0500	11.19	52.2	70.0	-3.39	94.94	F							1.09E+04																							
Koparin	C16H12O6	299.0551	11.85	38.7	9.8	-3.41	87.57	F																											8.18E+03		6.31E+03	
Violanone	C17H16O6	315.0857	12.19	38.5	9.4	-5.35	89.36	F																	2.40E+03				3.40E+02				9.22E+03				3.99E+03	

m /z = mass/charge; RT = retention time; FS = fragmentation score; IS = isotope similarity; F = flavonoids; PA = phenolic acids; OP = other polyphenols; F = free extract; B = bound extract T = two extracts. Bold represents reference standards.

Supplementary Table 2. List of phenolic compounds grouped according to the hierarchical cluster analysis (HCA) presented in Figure 4.

Compound name	Class	*m/z*	RT (min)	10DAF	17DAF	25DAF	33DAF	40DAF
**Group 1 (n = 23)**								
Nortrachelogenin/7-Hydroxymatairesinol/Isohydroxymatairesinol	L	373.1299	0.60	7.65E+02	0.00E+00	0.00E+00	0.00E+00	0.00E+00
Ethyl gallate	PA	197.0445	2.10	2.94E+02	0.00E+00	0.00E+00	0.00E+00	0.00E+00
Caffeoylquinic acid isomer I	PA	353.0868	4.14	2.40E+03	0.00E+00	0.00E+00	0.00E+00	0.00E+00
3,4-Dihydroxyphenylglycol	OP	169.0497	4.21	0.00E+00	8.84E+02	0.00E+00	0.00E+00	0.00E+00
**2,5-Dihydroxybenzoic acid**	**PA**	**153.0186**	**5.05**	4.93E+04	0.00E+00	0.00E+00	0.00E+00	0.00E+00
Caffeoylquinic acid isomer II	PA	353.0867	5.46	8.34E+02	0.00E+00	0.00E+00	0.00E+00	0.00E+00
Paeonol	OP	165.0551	5.55	0.00E+00	1.12E+03	0.00E+00	0.00E+00	0.00E+00
p-Coumaric acid 4-O-glucoside	PA	325.0915	5.82	6.26E+02	0.00E+00	0.00E+00	0.00E+00	0.00E+00
5-Caffeoylquinic acid	PA	353.0865	5.87	8.02E+04	1.54E+04	3.27E+04	2.58E+04	4.27E+04
3-Feruloylquinic acid	PA	367.1023	6.07	4.95E+04	4.73E+04	1.10E+04	4.46E+04	3.02E+04
Feruloyl glucose	PA	355.1023	6.46	2.51E+03	1.89E+04	2.44E+04	4.16E+03	1.28E+04
Caffeoylquinic acid isomer III	PA	353.0863	6.64	2.82E+04	0.00E+00	0.00E+00	0.00E+00	0.00E+00
Feruloylquinic acid isomer	PA	367.1024	6.96	1.06E+03	0.00E+00	0.00E+00	0.00E+00	0.00E+00
**4-Hydroxyphenylacetic acid**	**PA**	**151.0393**	**7.16**	3.71E+02	0.00E+00	0.00E+00	0.00E+00	0.00E+00
Dihydroquercetin 3-O-rhamnoside	F	449.1080	7.32	0.00E+00	1.13E+03	0.00E+00	0.00E+00	0.00E+00
Eriodictyol	F	287.0557	8.00	5.28E+02	0.00E+00	0.00E+00	0.00E+00	0.00E+00
**Quercetin**	**F**	**301.0347**	**8.01**	3.73E+02	0.00E+00	0.00E+00	0.00E+00	0.00E+00
**Sinapic acid**	**PA**	**223.0602**	**8.09**	3.15E+02	0.00E+00	0.00E+00	0.00E+00	0.00E+00
Phloridzin	F	435.1281	8.55	5.01E+02	6.98E+02	0.00E+00	0.00E+00	0.00E+00
Isorhamnetin 3-O-rutinoside	F	461.1071	8.59	5.53E+02	2.20E+03	0.00E+00	0.00E+00	0.00E+00
Neoeriocitrin	F	595.1658	8.69	4.39E+02	0.00E+00	0.00E+00	0.00E+00	0.00E+00
Trihydroxyisoflavanone isomer	F	271.0600	9.59	4.38E+02	0.00E+00	0.00E+00	0.00E+00	0.00E+00
NI	NI	191.0343	10.16	5.45E+02	0.00E+00	0.00E+00	0.00E+00	0.00E+00
**Group 2 (n = 21)**								
Dihydroformononetin	F	269.0835	0.55	0.00E+00	0.00E+00	1.26E+03	1.95E+03	0.00E+00
Danshensu	PA	197.0448	3.10	3.33E+04	5.94E+04	1.66E+05	2.53E+05	0.00E+00
**(+)-Catechin**	**F**	**289.0710**	**5.91**	9.35E+04	1.68E+05	6.62E+05	6.78E+05	0.00E+00
**4-Hydroxybenzaldehyde**	**OP**	**121.0289**	**6.14**	5.50E+05	8.20E+05	1.84E+06	1.66E+06	2.83E+05
4-Hydroxymandelic acid	PA	167.0342	6.14	7.98E+03	1.18E+04	2.95E+04	2.91E+04	0.00E+00
Dihydroquercetin	F	303.0505	6.34	0.00E+00	0.00E+00	1.31E+03	0.00E+00	0.00E+00
4-p-Coumaroylquinic acid	PA	337.0917	6.85	1.85E+03	0.00E+00	1.19E+03	7.42E+03	0.00E+00
Dihydroxybenzoic acid isomer II	PA	153.0187	7.05	9.52E+02	1.69E+03	5.95E+03	6.14E+05	0.00E+00
2,3,4-trihydroxybenzoic acid	PA	169.0134	7.05	0.00E+00	0.00E+00	0.00E+00	9.59E+03	0.00E+00
Cinnamoyl glucose	PA	309.0998	7.12	0.00E+00	0.00E+00	1.33E+03	1.97E+03	0.00E+00
Eriodictyol 7-O-glucoside	F	449.1081	7.50	0.00E+00	0.00E+00	2.13E+03	0.00E+00	0.00E+00
Luteolin 7-O-rutinoside	F	593.1497	8.11	3.10E+03	2.56E+03	1.56E+04	1.67E+04	0.00E+00
Glycitein	F	283.0601	8.43	0.00E+00	0.00E+00	0.00E+00	3.56E+03	0.00E+00

Hesperetin	F	301.0710	9.01	6.47E+03	0.00E+00	3.31E+04	0.00E+00	0.00E+00
Procyanidin dimer B-type V	F	577.1548	9.01	0.00E+00	0.00E+00	0.00E+00	2.67E+04	7.69E+01
Homoeriodictyol	F	301.0702	9.64	0.00E+00	0.00E+00	0.00E+00	6.27E+03	0.00E+00
Sakuranetin	F	285.0757	9.89	0.00E+00	0.00E+00	0.00E+00	1.08E+04	0.00E+00
1-Acetoxypinoresinol	L	415.1394	10.50	0.00E+00	4.78E+03	9.91E+03	1.08E+04	0.00E+00
Jaceosidin	F	329.0655	10.96	9.53E+02	1.47E+03	0.00E+00	6.28E+03	0.00E+00
Nepetin	F	315.0500	11.19	0.00E+00	0.00E+00	0.00E+00	1.09E+04	0.00E+00
Violanone	F	315.0857	12.19	3.40E+02	0.00E+00	9.22E+03	2.40E+03	3.99E+03
**Group 3 (n = 53)**								
Hesperidin	F	609.1881	0.57	1.31E+04	3.65E+04	5.12E+04	6.34E+04	6.86E+04
Catechol	OP	109.0289	0.94	1.29E+03	2.60E+03	3.07E+03	8.17E+03	1.15E+04
Dihydroxybenzoic acid isomer I	PA	153.0186	3.46	1.75E+04	3.50E+04	6.34E+04	1.83E+05	2.33E+05
3,4-Dihydroxyphenylacetic acid	PA	167.0343	3.80	0.00E+00	5.32E+03	7.34E+03	1.18E+04	1.25E+04
Methylcatechol isomer	OP	123.0445	4.21	0.00E+00	2.14E+03	2.29E+03	3.69E+03	3.19E+03
Dihydrocaffeic acid	PA	181.0500	4.46	6.56E+04	1.23E+05	2.74E+05	3.84E+05	3.47E+05
Homovanillic acid	PA	181.0499	4.73	1.36E+04	2.59E+04	0.00E+00	7.82E+04	6.69E+04
**4-Hydroxybenzoic acid**	**PA**	**137.0237**	**5.03**	2.22E+04	4.23E+04	8.06E+04	1.94E+05	1.93E+05
Procyanidin dimer B-type I	F	577.1338	5.42	2.12E+04	3.50E+04	1.63E+05	1.86E+05	1.25E+05
Procyanidin trimer C-type I	F	865.1963	5.76	1.23E+04	2.04E+04	9.39E+04	1.11E+05	9.14E+04
**Vanillin**	**OP**	**151.0393**	**5.80**	5.48E+03	4.07E+03	8.14E+03	4.82E+04	6.30E+04
Coumaroyl Hexoside	PA	325.0910	5.96	7.78E+02	3.48E+03	0.00E+00	7.11E+03	1.55E+04
Esculetin	OP	177.0185	6.10	1.10E+04	2.38E+04	5.01E+04	4.49E+05	6.66E+05
Procyanidin trimer C-type II	F	865.1956	6.14	1.03E+03	1.57E+03	8.07E+03	1.08E+04	6.29E+03
**Caffeic acid**	**PA**	**179.0343**	**6.24**	1.26E+05	1.54E+05	1.81E+05	1.37E+06	1.50E+06
Phenylacetic acid	PA	135.0444	6.51	3.73E+02	0.00E+00	0.00E+00	1.07E+04	6.49E+03
Umbelliferone	OP	161.0236	6.60	0.00E+00	0.00E+00	0.00E+00	6.12E+03	5.04E+03
Eriodictyol 7-O-glucoside	F	449.1073	6.95	2.65E+03	5.41E+03	2.07E+04	3.57E+04	2.96E+04
**p-coumaric acid**	**PA**	**163.0393**	**7.48**	4.24E+04	2.83E+05	2.28E+05	6.73E+05	7.80E+05
Procyanidin dimer B-type III	F	577.1335	7.48	4.13E+03	6.10E+03	2.15E+04	2.02E+04	1.55E+04
**trans-ferulic acid**	**PA**	**193.0499**	**8.00**	1.63E+05	6.36E+05	6.30E+05	1.00E+06	8.88E+05
Quercetin 3-O-rutinoside	F	609.1447	8.07	5.30E+03	7.21E+03	1.50E+04	1.48E+04	1.45E+04
Tetramethoxyflavone isomer I	F	341.1019	8.30	4.49E+03	1.50E+04	1.12E+04	3.68E+04	3.54E+04
Naringin 4'-O-glucoside	F	433.1128	8.35	2.82E+03	1.46E+04	3.73E+04	4.47E+04	3.79E+04
Morin	F	301.0343	9.80	8.09E+02	1.22E+03	1.21E+03	2.93E+03	3.96E+03
Dihydroxy-trimethoxyflavone isomer II	F	343.0811	9.93	3.72E+03	1.01E+04	2.48E+04	4.27E+04	2.61E+04
Puerarin	F	415.1027	9.94	0.00E+00	1.18E+03	0.00E+00	5.36E+06	5.62E+06
Glycitin	F	445.1126	10.55	0.00E+00	0.00E+00	0.00E+00	3.23E+05	2.21E+05
Isorhamnetin	F	315.0501	10.92	0.00E+00	0.00E+00	0.00E+00	9.47E+03	1.47E+04
Koparin	F	299.0551	11.85	0.00E+00	0.00E+00	0.00E+00	8.18E+03	6.31E+03
Vanillactic acid	PA	211.0608	4.25	0.00E+00	0.00E+00	0.00E+00	0.00E+00	4.55E+03
Ferulaldehyde	OP	177.0185	5.58	1.34E+03	2.08E+03	6.58E+03	0.00E+00	1.37E+04
Methoxyphenylacetic acid	PA	165.0550	6.00	2.86E+02	3.06E+03	0.00E+00	0.00E+00	8.34E+03
p-anisaldehyde	OP	135.0444	7.07	1.35E+03	1.43E+04	0.00E+00	0.00E+00	1.72E+05

Naringenin 7-O-glucoside	F	433.1128	7.84	1.33E+04	6.32E+04	1.66E+05	1.47E+05	1.97E+05
Ferulic acid	PA	193.0498	8.25	7.55E+04	1.71E+05	2.71E+05	2.54E+05	4.03E+05
NI	F	771.1914	8.30	0.00E+00	2.49E+03	3.31E+03	1.83E+03	4.26E+03
6-hydroxyluteolin	F	301.0343	8.35	1.26E+03	0.00E+00	0.00E+00	0.00E+00	8.54E+03
Chrysoeriol 7-O-apiosyl-glucoside	F	593.1497	8.53	4.26E+02	7.86E+02	0.00E+00	0.00E+00	2.67E+03
Tetramethoxyflavone isomer II	F	341.1018	8.67	1.36E+03	3.59E+03	9.19E+03	7.64E+03	1.40E+04
Isorhamnetin 3-O-glucoside	F	477.1026	8.85	1.00E+03	1.81E+03	7.29E+03	1.05E+04	2.57E+04
Apigenin 6-C-glucoside	F	431.0976	8.87	0.00E+00	0.00E+00	0.00E+00	0.00E+00	1.31E+04
Tectoridin	F	461.1072	9.04	0.00E+00	0.00E+00	0.00E+00	0.00E+00	3.36E+03
Dihydroxy-trimethoxyflavone isomer I	F	343.0811	9.05	0.00E+00	1.78E+03	3.28E+03	4.66E+03	1.26E+04
Procyanidin dimer B-type VI	F	577.1341	9.43	4.57E+02	9.60E+02	0.00E+00	0.00E+00	8.13E+03
Rhamnetin	F	315.0500	9.55	8.55E+02	0.00E+00	0.00E+00	0.00E+00	6.46E+03
Tetramethoxyflavone isomer III	F	341.1019	10.12	1.04E+04	2.30E+04	3.29E+04	3.88E+04	5.59E+04
Scutellarein	F	285.0392	10.16	8.49E+02	8.40E+04	1.88E+05	4.09E+05	8.51E+05
Procyanidin dimer B-type VII	F	577.1334	10.26	4.26E+02	1.32E+03	4.91E+03	0.00E+00	1.45E+04
Coumestrol	OP	267.0289	10.36	0.00E+00	0.00E+00	0.00E+00	0.00E+00	2.24E+03
Dihydroxy-trimethoxyflavone isomer III	F	343.0812	10.59	4.29E+02	1.96E+03	6.73E+03	1.12E+04	3.08E+04
Procyanidin dimer B-type VIII	F	577.1333	10.71	0.00E+00	0.00E+00	1.34E+03	0.00E+00	3.29E+03
3'-Hydroxymelanettin	F	299.0551	11.03	1.40E+04	3.21E+04	2.26E+05	3.35E+05	6.93E+05

m/z = mass/charge; RT = retention time; F = flavonoids; PA = phenolic acids; OP = other polyphenols; DAF = days after flowering. Bold represent reference standards.
